# Magnetically Separable Chiral Poly(ionic liquid) Microcapsules Prepared Using Oil-in-Oil Emulsions

**DOI:** 10.3390/polym16192728

**Published:** 2024-09-26

**Authors:** Reema Siam, Abeer Ali, Raed Abu-Reziq

**Affiliations:** Casali Center of Applied Chemistry, Institute of Chemistry, Center for Nanoscience and Nanotechnology, The Hebrew University of Jerusalem, Jerusalem 9190401, Israel; reema.siam@mail.huji.ac.il (R.S.); abeer.ali@mail.huji.ac.il (A.A.)

**Keywords:** magnetite, chiral poly(ionic liquid), polyurea microcapsules

## Abstract

This article presents a method for producing chiral ionic liquid-based polyurea microcapsules that can be magnetically separated. The method involves entrapping hydrophilic magnetic nanoparticles within chiral polyurea microspheres. The synthetic process for creating these magnetic polyurea particles involves oil-in-oil (o/o) nano-emulsification of an ionic liquid-modified magnetite nanoparticle (MNPs-IL) and an ionic liquid-based diamine monomer, which comprises a chiral bis(mandelato)borate anion, in a nonpolar organic solvent, toluene, and contains a suitable surfactant. This is followed by an interfacial polycondensation reaction between the isocyanate monomer, polymethylenepolyphenyl isocyanate (PAPI 27), and the chiral diamine monomer, which generates chiral polyurea microcapsules containing magnetic nanoparticles within their cores. The microcapsules generated from the process are then utilized to selectively adsorb either the R or S enantiomer of tryptophan (Trp) from a racemic mixture that is dissolved in water, in order to evaluate their chiral recognition capabilities. During the experiments, the magnetically separable chiral poly(ionic liquid) microcapsules, which incorporated either the R or S isomer of chiral bis(mandelato)borate, exhibited exceptional enantioselective adsorption performance. Thus, the chiral polymeric microcapsules embedded with the R-isomer of the bis(mandelato)borate anion demonstrated significant selectivity for adsorbing L-Trp, yielding a mixture with 70% enantiomeric excess after 96 h. In contrast, microcapsules containing the S-isomer of the bis(mandelato)borate anion preferentially adsorbed D-Trp, achieving an enantiomeric excess of 73% after 48 h.

## 1. Introduction

Chiral molecules have a broad range of applications [1], which has led to significant efforts being made in developing methods for the enantioselective synthesis, separation, and isolation of these molecules [2,3,4]. Enantiomerically pure compounds are of great importance in the pharmaceutical and agrochemical industries, as the biological activity of chiral drugs can differ greatly depending on the target species [5,6,7,8,9]. One enantiomer may exhibit the desired biological effects, while the other optical isomer can be inactive or even toxic [10]. It is therefore essential to identify and utilize the correct enantiomer in order to achieve the desired therapeutic effect. Recently, there has been a growing interest in the construction of chiral solid materials and surfaces, as they have the potential to be utilized in chiral adsorption, enantioseparation, and catalysis [11,12,13,14,15,16,17,18,19,20]. Chiral polymeric particles are a significant category of chiral solids that have gained considerable interest in various fields such as drug delivery, catalysis, sensors, and chiral separation due to their unique characteristics [21,22,23,24,25,26]. These particles exhibit intriguing properties such as selective adsorption, chiral recognition, and optical activity because of their chiral nature [24]. Several techniques have been devised to create chiral polymeric particles, including emulsion polymerization, miniemulsion polymerization, and dispersion polymerization [24].

Poly(ionic liquid)s (PILs) are a distinct class of functional polyelectrolytes, in which each repeating unit carries an ionic liquid (IL) species. These PILs have garnered growing interest in the fields of polymer chemistry and materials science, owing to the exceptional properties resulting from their combination of ionic liquid and macromolecular architecture, such as processability, durability, mechanical strength, and thermal stability [27,28,29,30,31]. This unusual blend of properties makes PILs highly attractive for various applications, such as electrochemical devices, catalysis, and separation [32,33,34,35,36,37]. There are two main approaches to synthesizing PILs [28,29,30]. The first method involves the direct polymerization of ionic liquid monomers, while the second method involves modifying pre-existing polymers through quaternization or ion metathesis. Recently, due to their unique properties and wide range of potential applications, various techniques have been developed for the preparation of poly(ionic liquid) particles (PILPs) [28,38]. The methods employed for PILP synthesis are largely based on emulsion polymerization and suspension polymerization, which are selected based on the desired particle size, morphology, and characteristics.

While there is extensive research on polymeric ionic liquids, the investigation of their chiral counterparts is still uncommon [28]. Chiral poly(ionic liquid)s (CPILs) are polymers that contain both chiral and ionic components, which gives rise to their unique properties. CPILs have demonstrated promise as materials for use in asymmetric catalysis, sensors, and enantioselective separation applications. For example, CPILs were synthesized by introducing optically pure amino acids into polymeric ionic liquids (PILs) via an anionic exchange reaction [39]. The CPILs showed tunable chirality and induced helicity, as observed through various analyses. The chiral PILs were used as catalysts in the Baylis–Hillman reaction, giving a high yield of the desired product with moderate enantiomeric excess. Wang et al. developed a template-free and one-step method to create chiral mesoporous poly(ionic liquids) using a Friedel–Crafts hyper-crosslinking reaction [40]. They utilized a chiral manganese (III) salen complex to produce a chiral polymeric ionic liquid which demonstrated high catalytic activity and enantioselectivity in the asymmetric epoxidation of unfunctionalized olefins. Wu et al. developed a novel fluorescent chiral poly(ionic liquid) through micelle-controlled free-radical polymerization [41]. The polymer has a nonconjugated backbone and shows photoluminescence via spatial π−π and ion−π interactions. It can serve as a chiral sensor for phenylalaninol and tryptophan detection in the presence of Cu(II), exhibiting distinct fluorescence responses. Recently, a new synthetic strategy to produce chiral porous polymer membranes by crosslinking chiral poly(ionic liquid)s with water via hydrogen bonding has been developed [42]. The resulting chiral polymeric membranes showed efficient enantioselective separation of penicillamine enantiomers via directional H-bonding interactions.

In this work, we have developed a new method for producing chiral polymeric ionic liquid microcapsules with magnetic properties for enantioselective adsorption. The method involves interfacial polymerization of a chiral ionic liquid-based diamine monomer with a polyisocyanate in an oil-in-oil emulsion. The chiral poly(ionic liquid) microcapsules obtained exhibited effective performance in enantioselective adsorption of tryptophan.

## 2. Materials and Methods

### 2.1. General Information

An FTIR 65 spectrometer from Perkin Elmer was used to record the infrared spectra. Scanning electron microscopy (SEM) was performed on a Sirion SEM microscope (FEI Company, USA) equipped with a Shottky-type emission source and a secondary electron detector, running at a voltage of 5 kV. Transmission electron microscopy (TEM) was conducted using a TEM Tecnai F20 G2 (FEI Company, USA) operating at 200 kV. The Nano Series instrument, model Nano-Zetasizer ZEN3600 (Malvern Instruments, UK), was utilized for determining size distribution and zeta potential. Thermogravimetric analysis (TGA) was carried out on a Mettler Toledo TG 50 analyzer (Mettler Toledo, Switzerland) within a temperature range of 25 to 900 °C, under an inert atmosphere (N_2_), and with a heating rate of 10 °C/min. Circular dichroism (CD) analyses were performed on a MOS 500 spectrometer (BioLogic instruments, France) under a xenon lamp source, using Biokine software (version 1.53.0.1). ^1^H NMR and ^13^C NMR spectra were recorded with Bruker DRX-400 and DRX-500 instruments (Bruker, Germany). The magnetite content in the chiral microcapsules was determined using inductively coupled plasma (ICP) analysis with an Agilent 8900 triple quadrupole ICP-MS system (Agilent, Japan). Magnetic measurements were conducted using a Lake Shore 8600 Series vibrating sample magnetometer (VSM) (LakeShore Cryotronics, USA).

### 2.2. Preparation of 3-Butyl-1-[3-(Trimethoxysilyl)Propyl]-1H-Imidazol-3-Ium Chloride (IL-C4) [43]

3-Chloropropyltrimethoxysilane (21.1 mL, 114.3 mmol) and 1-butylimidazole (15 mL, 114.3 mmol) were stirred under nitrogen at 120 °C for 72 h. The mixture was cooled down to room temperature and a yellow-orange viscous liquid was obtained. ^1^H NMR (400 MHz, CDCl_3_): δ (ppm) = 0.02 (t, J = 8.0 Hz, 3H), 0.299 (t, J = 7.51 Hz, 3H), 0.72 (m, 2H), 1.28 (m, 2H), 1.37 (m, 2H), 2.9 (s, 9H), 3.74 (m, 4H), 7.02 (t, J = 1.83, 1H), 7.24 (t, J = 1.83, 1H), 9.93 (s, 1H)^13^C NMR (100 MHz, CDCl_3_): δ (ppm) = 5.49, 13.0, 18.98, 23.7, 31.7, 49.1, 50.2, 51.1, 121.8, 122.3, 136.7.

### 2.3. Preparation of Magnetite Nanoparticles Modified with IL-C4 (MNPs-IL-C4) [43]

To prepare magnetite nanoparticles, 11.7 g FeCl_3_.6H_2_O and 4.3 g FeCl_2_.4H_2_O were dissolved in 400 mL of deionized water, and mechanically stirred under nitrogen. The mixture was heated to 90 °C and 18 mL of concentrated ammonia (25%) was quickly added, resulting in a black suspension of magnetite nanoparticles. The mixture was further heated for 15 min before being cooled to room temperature. The black precipitate was separated using an external magnetic field and washed four times with 250 mL of deionized water and once with 200 mL of ethanol. The magnetite nanoparticles were then suspended in 400 mL of ethanol and sonicated for 90 min. After that, a solution of 100 mL of ethanol containing 30 mmol of IL-C4 was added, followed by the addition of 15 mL of concentrated ammonia (25%). The reaction mixture was stirred under nitrogen for 36 h at room temperature. The modified magnetite nanoparticles were magnetically separated and washed three times with ethanol and once with methanol. They were then suspended in 400 mL of methanol and mechanically stirred for an additional 3 h at room temperature. After adding 100 mL of diethyl ether, the modified magnetite nanoparticles were magnetically separated, washed twice with diethyl ether, and dried under vacuum for 12 h. A black powder of magnetite nanoparticles (MNPs-IL-C4) weighing 4.7 g was obtained.

### 2.4. Preparation of Tert-Butyl N-(3-Chloropropyl)Carbamate [44]

Triethylamine (8.8 mL, 63.1 mmol) was added slowly to a solution of 3.9 g (30 mmol) 3-chloropropylamine hydrochloride in 60 mL dichloromethane, and the resulting mixture was stirred for 30 min at 0 °C. Then, 7.20 g (33 mmol) of di-*tert*-butyl dicarbonate was added and the mixture stirred for 5 min at 0 °C and at room temperature for 21 h. Next, 100 mL of distilled water was added to the mixture, followed by the addition of 100 mL of chloroform. After separation of the organic phase, the aqueous phase was extracted twice with 100 mL of chloroform. The combined organic phases were dried with MgSO_4_; the solution was concentrated by the evaporator to yield 5.6 g of an oily product. ^1^H NMR (400 MHz, CDCl_3_): δ (ppm) = 1.42 (s, 9H), 1.91–1.98 (m, 2H), 3.25 (t, J = 6.94 Hz, 2H), 3.56 (t, J = 6.25 Hz, 2H); ^13^C NMR (100 MHz, CDCl_3_): δ (ppm) = 28.3, 31.2, 32.6, 38.0, 42.3, 79.3, 156.0.

### 2.5. Preparation of Tert-Butyl (3-(1H-Imidazol-1-Yl)Propyl)Carbamate [45]

A solution of 4.2 g of NaHCO_3_ in 50 mL water was added to a solution of 1-(3-aminopropyl)imidazole (2.4 mL, 20.1 mmol) in 50 mL tetrahydrofuran (THF) at 0 °C. Then, to the resulting mixture, a solution of di-*tert*-butyl dicarbonate (5.7 g, 26.1 mmol) in 25 mL of THF was added dropwise while stirring. The mixture was stirred for 3 h at room temperature, and after removing the solvent, the residue was dissolved in ethyl acetate and washed with water three times. The organic phase was dried with MgSO_4_. The solvent was evaporated to give 3.6 g of an oily product. ^1^H NMR (400 MHz, CDCl_3_): δ (ppm) = 1.37 (s, 9H), 1.86–1.93 (m, 2H), 3.06 (bs, 2H), 3.92 (t, J = 7.03 Hz, 2H), 4.9 (bs, NH), 6.8 (s, 1H), 6.9 (s, 1H), 7.4 (s, 1H); ^13^C NMR (100 MHz, CDCl_3_): δ (ppm) = 28.3, 31.6, 37.6, 44.3, 118.8, 129.5, 137.1, 156.1.

### 2.6. Preparation of 1,3-Bis(3-((Tert-Butoxycarbonyl)Amino)Propyl)-1H-Imidazol-3-Ium Chloride (BAPIC) [46]

*Tert*-butyl (3-(1*H*-imidazol-1-yl)propyl)carbamate (4.78 g, 21.23 mmol) and *tert*-butyl N-(3-chloropropyl)carbamate (3.88 g, 20 mmol) were dissolved in 50 mL toluene and refluxed under nitrogen for 98 h. After the mixture was cooled to room temperature, an oil phase was formed at the bottom of the flask, which was separated by decantation of the toluene. Then, the oily product was washed with diethyl ether one time and twice with ethyl acetate and then all residual solvents were removed by evaporation. ^1^H NMR (400 MHz, D_2_O): δ (ppm) = 1.35 (s, 18H), 1.98–2.04 (m, 4H), 3.05 (t, J = 6.29 Hz, 4H), 4.19 (t, J = 6.89 Hz, 4H), 7.48 (s, 2H), 8.74 (s, 1H); ^13^C NMR (100 MHz, D_2_O): δ (ppm) = 27.7, 29.2, 36.4, 47.1, 81.0, 122.5, 135.5, 158.1.

### 2.7. Preparation of 1,3-Bis(3-Aminopropyl)-1H-Imidazol-3-Ium-2,7-Dioxo-3,8-Diphenyl-1,4,6,9-Tetraoxa-5-Boraspiro [4.4] Nonan-5-Uide (IL-NH_2_-BMB)

In a mixture of 0.231 g (3.12 mmol) of lithium carbonate and 0.388 g (6.27 mmol) of boric acid in 6 mL of water, 1.83 g (12 mmol) of either *R*-mandelic acid or *S*-mandelic acid was slowly added to form the proper bis(mandelato)borate anion, [B(*R*-Man)_2_]^-^ or [B(*S*-Man)_2_]^-^. The solution was heated to 55 °C for an hour, then cooled to room temperature and 0.60 g (1.43 mmol) of BAPIC was added. After an hour of stirring, 40 mL of dichloromethane was added to extract the desired product. The organic layer was washed four times with 3 mL of water. After evaporating the dichloromethane, 50 mL of water was added and the resulting mixture was refluxed for 48 h to remove the *tert*-butyloxycarbonyl (Boc) group. The aqueous solution was then washed three times with 50 mL of ethyl acetate, and the water was evaporated to obtain the desired ionic liquid monomer, IL-NH_2_-BMB. Finally, the product was dried under vacuum (2.3 mmHg) at 60 °C overnight. ^1^H NMR (400 MHz, DMSO-d6): δ (ppm) = 1.87 (quint, 1.87, J = 5.35 Hz, 4 H), 2.54 (t, J = 5.12 Hz, 4H), 4.23 (t, J = 5.2 Hz, 4H), 5.15 (bs, 1H), 5.18 (bs, 1H), 7.30 (t, J= 5.76 Hz, 2H), 7.36 (t, J= 5.84 Hz, 4H), 7.36 (t, J= 5.84 Hz, 4H), 7.55 (dd, J= 13.7, 6 Hz, 4H), 7.78 (s, 2H), 9.19 (s, 1H); ^13^C NMR (100 MHz, DMSO-d6): δ (ppm) = 33.88, 39.11, 48.0, 78.42, 78.55, 123.80, 127.41, 127.44, 127.49, 128.50,128.54, 129.19, 129.23, 129.24, 137.46, 141.80, 142.0, 177.93, 177.97.

### 2.8. Preparation of Magnetically Separable Chiral Polyurea Microcapsules

The chiral polyurea microcapsules were prepared using the interfacial polymerization of an oil-in-oil emulsion technique. Firstly, a non-polar phase was formed by dissolving a surfactant solution (Agrimer Al-22 or ABIL-EM90) in 90 g of toluene at different concentrations (ranging from 1% to 5% *w*/*w*) and then homogenized at 10,000 rpm for 30 s. Next, a polar phase was formed by mixing 4.65 g of polar organic solvent, 0.02–0.2 g of MNPs-IL-C4, and chiral IL-NH_2_-BMB (0.6 g, 1.6 mmol); it was then sonicated for 2 h. This polar phase was rapidly added to the non-polar phase during homogenization, and the emulsification process was carried out for a further 1.5 min at 10,000 pm, followed by sonication for 10 min using an ultrasonic cell disrupter with an output of 130 Watt and 20 kHz. Finally, 0.55 g of PAPI 27 dissolved in 9.45 g of toluene was added rapidly to the emulsion system during sonication, and the resulting emulsion was mechanically stirred at room temperature for 24 h. The chiral PU particles were collected using a magnet and washed three times with toluene before being re-suspended in toluene. The magnetite content in the chiral microcapsules was analyzed using ICP. For this, 10 mg of chiral microcapsules were mixed with 0.267 mL of 36% HCl in a 10 mL volumetric flask. After 30 min of sonication, the mixture was diluted to 10 mL using triple-distilled water.

### 2.9. Adsorption of Tryptophan (Trp) on the Magnetic Chiral Polyurea Microcapsules

20 mL of a DL-tryptophan (Trp) solution with a concentration of 0.49 mM in water was added to 0.2 g of the chiral polymeric microcapsules containing within their shells R- or S-isomer of bis(mandelato)borate anion. The resulting solution was subjected to circular dichroism (CD) analysis at 4, 8, 12, 24, 48, 72, and 96 h to determine the percentage of the specific enantiomer of Trp that was adsorbed. The chiral microcapsules were isolated from the solution using an external magnetic field, washed three times with 20 mL of water, and subsequently reused for the enantioseparation of DL-tryptophan.

## 3. Results and Discussion

### 3.1. Preparation and Characterization of Magnetite Nanoparticles Modified by Ionic Liquid (MNPs-IL-C4)

**Figure 1:**
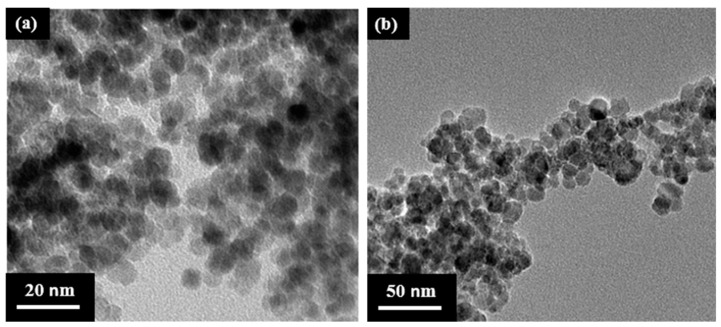
TEM images of (**a**) bare magnetic nanoparticles (MNPs) and (**b**) MNPs-IL-C4.

**Scheme 1:**
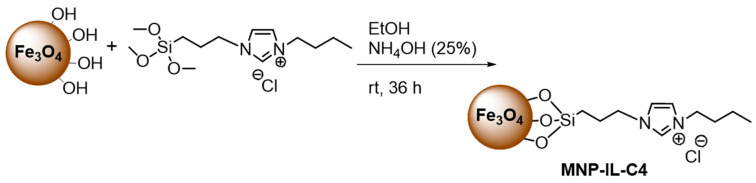
Preparation of magnetite nanoparticles modified with an ionic liquid group.

MNPs were produced using Massart’s method, which involves co-precipitating FeCl_2_ and FeCl_3_ in a basic aqueous medium with ammonia solution at 85–90 °C, under an inert atmosphere. The resulting MNPs were spherical and had a size range of 5–20 nm, as confirmed by transmission electron microscopy (TEM) analysis (Figure 1a). However, these MNPs were found to be unstable and tended to aggregate. Therefore, it was necessary to stabilize them by modifying their surface. To achieve this, the MNPs were covalently bonded with an ionic liquid, 1-butyl-3-(3-(trimethoxysilyl) propyl)-1*H*-imidazol-3-chloride (IL-C4), through the condensation of the trimethoxysilyl groups of IL-C4 with the surface hydroxyl groups of the MNPs (as shown in Scheme 1). According to TEM analysis (Figure 1b), the size of the magnetic nanoparticles did not change after they were modified with the IL-C4. Infrared analysis (Figure S1 in the Supporting Information) verified the success of the modification process with IL-C4. This modification not only stabilized the MNPs but also improved their dispersibility in various polar solvents, including dimethyl sulfoxide (DMSO), dimethylformamide (DMF), N,N-dimethyl acetamide (DMAc), and water.

The dispersibility of the modified particles was assessed using dynamic light scattering (DLS). To determine the most effective solvent for dispersing the modified MNPs, we dispersed the particles in various solvents at different concentrations and observed the extent of particle aggregation, as indicated by the particle size distribution (Supporting Information, Figure S2). The best results were obtained when the MNPs-IL-C4 were dispersed in DMSO and water, in which the smallest particle size distribution was achieved (Supporting Information, Figure S2a,d). However, when dispersed in DMAc, two populations of particles were observed, one consisting of particles which were less aggregated and the other of larger aggregates.

### 3.2. Preparation and Characterization of a Chiral Ionic Liquid-Based Diamine Monomer (IL-NH_2_-BMB)

**Scheme 2:**
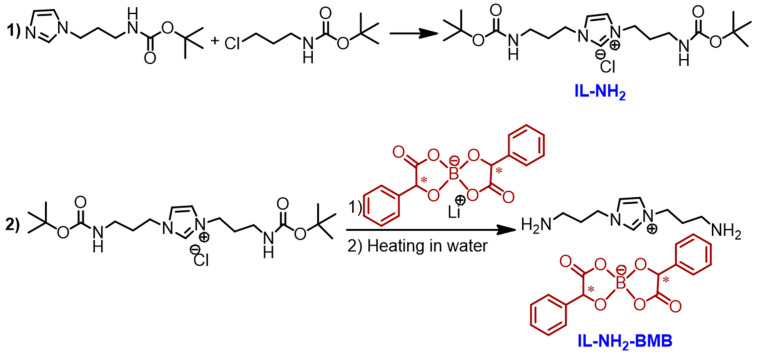
Preparation of a chiral ionic liquid-based diamine monomer (IL-NH_2_-BMB). The asterisk (*) refers to a chiral center.

In order to prepare chiral polyurea particles, our approach involved the utilization of a chiral diamine capable of reacting with achiral isocyanate monomers to form the polymeric shell. To achieve this, we developed a new ionic liquid-based diamine monomer in which the anion component was a chiral spiroborate. The synthesis of this monomer began with the production of 1,3-bis(3-aminopropyl)-1*H*-imidazol-3-ium chloride (IL-NH_2_, as illustrated in Scheme 2). To synthesize IL-NH_2_, we initiated the reaction by combining *tert*-butyl (3-(1*H*-imidazol-1-yl)propyl)carbamate with *tert*-butyl (3-chloropropyl)carbamate. This reaction yielded the desired compound, IL-NH_2_, which served as the precursor for the chiral ionic liquid-based diamine monomer (IL-NH_2_-BMB). To introduce chirality into the ionic liquid-based diamine monomer, we performed an ion exchange process. Specifically, we replaced the chloride anion in IL-NH_2_ with a chiral spiroborate anion. The chiral spiroborate anion was synthesized by reacting the appropriate enantiomer of mandelic acid with boric acid in the presence of lithium carbonate. This reaction resulted in the formation of the desired chiral spiroborate anion. Next, we subjected the resulting material containing IL-NH_2_-BMB to a heating process in water. The purpose of this step was to remove the protecting groups, specifically the *tert*-butyloxycarbonyl (Boc) groups, and obtain the free amine groups. This preparation method enabled us to obtain the chiral ionic liquid-based diamine monomer (IL-NH_2_-BMB) with the necessary reactivity and chirality for the subsequent synthesis of chiral polyurea particles.

**Figure 2:**
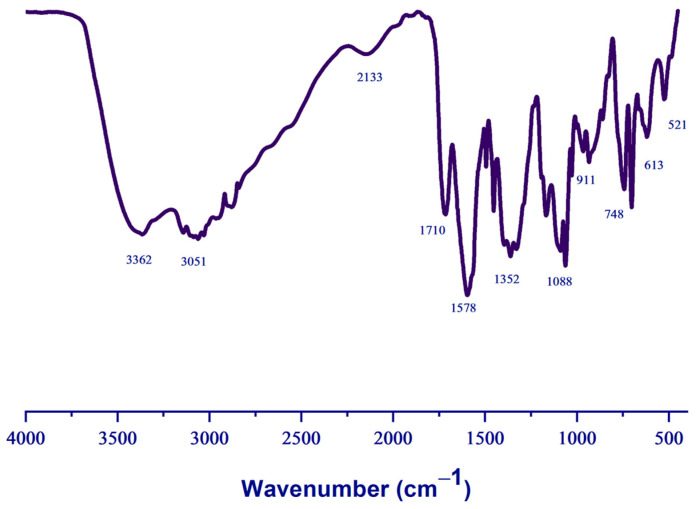
IR spectrum of the chiral IL-NH2-BMB.

The IL-NH_2_-BMB was characterized by various methods including IR spectroscopy, NMR, and circular dichroism (CD) spectroscopy. Infrared analysis revealed the presence of the functional groups of IL-NH_2_-BMB (Figure 2). Thus, the characteristic band peak at 3362 cm^−1^ belongs to N–H stretching bands, 1710 cm^−1^ is attributed to the carbonyl groups of the chiral spiroborate anion, and 1578 cm^−1^ is assigned to the characteristic N–H bending band of the primary amine groups.

A further indication of the formation of the chiral IL-NH_2_-BMB was obtained by ^1^H and ^13^C-NMR (Figures S18–S21). The ^1^H-NMR was performed separately in the solvents D_2_O and DMSO-d_6_ because one of peaks of IL-NH_2_-BMB overlaps with the peaks of DMSO. In D_2_O, the peak of the proton linked to the carbon C2 between the two nitrogen atoms in the imidazolium ring disappeared, most probably because of exchange process with D_2_O, which indicates the acidity of this proton (Figure S18). In DMSO-d_6_ this peak appeared clearly at 9.85 ppm (Figure S20). Both ^1^H and ^13^C-NMR (Figures S18–S21) analyses confirmed the obtaining of the expected structure of the prepared IL-NH_2_-BMB.

**Figure 3:**
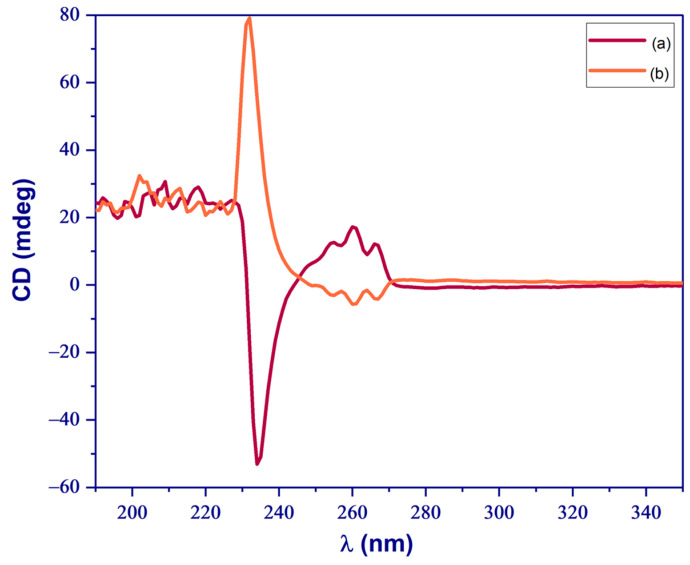
Circular dichroism (CD) spectrum of (a) IL-NH_2_-BMB containing R-isomer of bis(mandelato)borate anion, and (b) IL-NH_2_-BMB containing S-isomer of bis(mandelato)borate anion.

CD spectroscopy was utilized to ascertain the chirality of the synthesized IL-NH_2_-BMB, incorporating the chiral bis(mandelato)borate anion, as depicted in Figure 3. The analysis was carried out using a solution of IL-NH_2_-BMB dissolved in water at a concentration of 0.56 mM. The IL-NH_2_-BMB derived from the R-isomer of the bis(mandelato)borate anion exhibited a prominent negative signal at 234 nm, whereas the IL-NH_2_-BMB featuring the S-isomer of the bis(mandelato)borate anion displayed a notable positive signal at 230 nm. This spectroscopic analysis confirms the distinct chirality of the synthesized IL-NH_2_-BMB molecules based on their respective bis(mandelato)borate anion substituents.

### 3.3. Preparation and Characterization of Magnetically Separable Chiral Poly(ionic liquid) Microcapsules

**Scheme 3:**
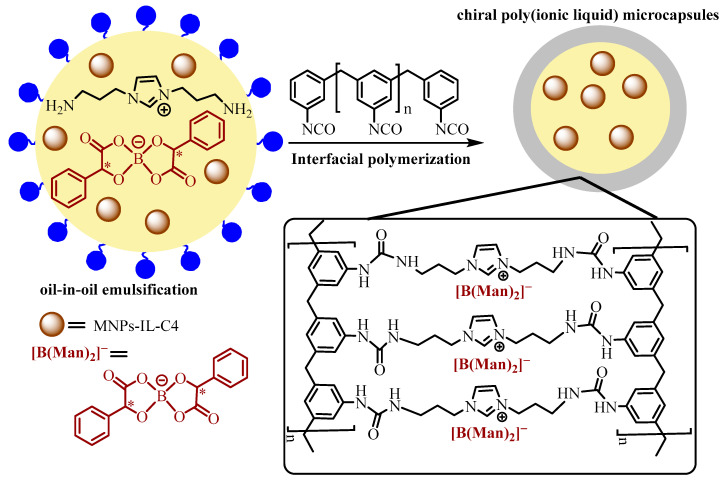
Preparation of chiral polyurea particles from the isocyanate monomer PAPI 27 and the chiral amine monomer IL-NH_2_-BMB. The asterisk (*) refers to a chiral center.

The chiral poly(ionic liquid) microcapsules were prepared from oil-in-oil (O/O) nanoemulsion via an interfacial polycondensation process of the isocyanate monomer, PAPI 27, and chiral IL monomer (Scheme 3). The NPs were fabricated by emulsifying the dispersed oil phase, comprising polar organic solvent, MNPs-IL-C4, and chiral IL-NH_2_-BMB, with a surfactant solution composed of toluene and the lipophilic polymeric a surfactant (Agrimer Al-22 or ABIL-EM90) solution of different ratio (1–5% *w*/*w*), followed by ultrasonication for 10 min. Subsequently, PAPI 27 dissolved in toluene was added quickly while sonicating the nanoemulsion system for 10 min. The interfacial polycondensation reaction between the isocyanate monomer and the chiral amine monomer occurred, creating magnetically separable polymeric particles with chiral shells.

To optimize the preparation of chiral polymeric microcapsules, we utilized a variety of polar organic solvents, different quantities of MNPs-IL-C4, and various surfactant types and ratios. Initially, our research focused on how the quantity of MNPs-IL-C4 influenced the formation of these chiral microcapsules across different solvents. Given that the dispersibility of modified magnetite nanoparticles varies with the solvent type, we anticipated that both the concentration of nanoparticles and the choice of solvent would impact the development of the desired chiral polymeric microcapsules. For these experiments, we utilized Agrimer AL22 (5%, *w*/*w*) and the R-isomer of IL-NH_2_-BMB, choosing Agrimer AL22 due to its effectiveness as a polymeric surfactant, as it is known to enhance the stability of oil-in-oil emulsions [47]. The findings from these experiments are summarized in Table 1.

We attempted to create chiral polymeric microcapsules using DMSO, DMF, and DMAc. The use of DMSO facilitated optimal dispersion of MNPs-IL-C4 up to 200 mg in 4.65 g of the solvent. TEM analysis confirmed successful microcapsule formation, although the encapsulation of magnetite nanoparticle varied with their concentrations. With up to 80 mg of magnetite nanoparticles, spherical microcapsules formed, with MNPs-IL-C4 detected mainly in part of the microcapsule cores (Table 1, entries 1–4). Increasing the magnetite amount to 200 mg resulted in a majority of microcapsules containing the nanoparticles within their cores (Table 1, entry 5). Under identical conditions, using the S-isomer of IL-NH_2_-BMB produced similar results, with most microcapsules containing the nanoparticles in their cores (Table 1, entry 6). When we switched to using DMF as the polar phase, we achieved good dispersion with MNPs-IL-C4 up to 80 mg. Using between 20 and 80 mg of magnetite nanoparticles led to the formation of chiral polymeric microcapsules, although only some contained the nanoparticles in their cores (Table 1, entries 7–9). Switching the polar solvent to DMAc, we managed to achieve homogeneous dispersion with MNPs-IL-C4 up to 160 mg. The formation of chiral polymeric microcapsules using DMAc as the polar phase in the oil-in-oil emulsion, with 20 to 160 mg of MNPs-IL-C4, resulted in microcapsules of which only some contained magnetite nanoparticles in their cores (Table 1, entry 10–12).

We experimented with ABIL EM 90 (5% *w*/*w*) as a surfactant instead of Agrimer AL 22, using DMSO as the polar solvent, along with 200 mg of MNPs-IL-C4 and the R-isomer of IL-NH_2_-BMB. The TEM analysis (Supporting Information, Figure S3) revealed the formation of microcapsules, but most of them lacked magnetite nanoparticles in their cores, as some particles were found outside the microcapsules. It appeared that this surfactant could not effectively stabilize the oil-in-oil emulsion when the polar phase contained magnetite nanoparticles, leading to the nanoparticles separating from the polar phase. This contrasted with Agrimer AL 22, which contains pyrrolidone groups in its polar segment and seemed to enhance the stability of MNPs-IL-C4 in the polar phase by coordinating with the surface of the magnetite nanoparticles.

Additionally, we varied the percentages of Agrimer AL22 to assess the effect of surfactant concentration on emulsion stability and, consequently, chiral polymeric microcapsule formation. In these tests, DMSO served as the polar solvent, with 200 mg of MNPs-IL-C4 and the R-isomer of IL-NH_2_-BMB. The results highlighted a notable impact of surfactant percentage: at 1% Agrimer AL 22, we could obtain polymeric microcapsules, but the magnetite nanoparticles tended to aggregate outside the capsules (Supporting Information, Figure S4a). Increasing the surfactant concentration to 2% resulted in fused microcapsules with magnetite nanoparticles aggregating outside (Supporting Information, Figure S4b). However, using 4% surfactant led to the formation of aggregated microcapsules containing magnetite nanoparticles in their cores (Supporting Information, Figure S4c).

**Figure 4:**
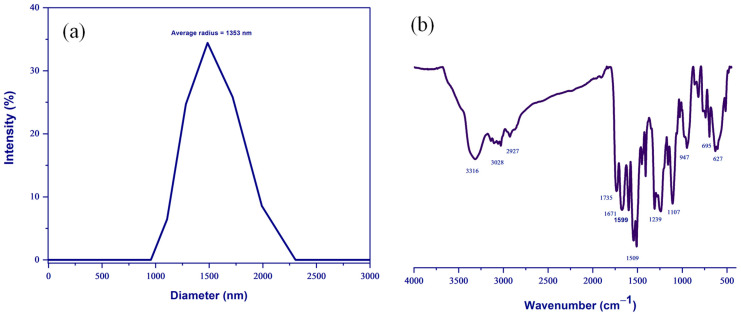
(**a**) Size distribution and (**b**) IR spectrum of magnetically separable chiral (polyionic liquid) microcapsules prepared using DMSO as polar solvent and the R-isomer of IL-NH_2_-BMB.

The chiral poly(ionic liquid) microcapsules, prepared using the optimal conditions, were subjected to comprehensive characterization, employing several analytical techniques including dynamic light scattering (DLS), zeta potential, scanning electron microscopy (SEM), transmission electron microscopy (TEM), infrared (IR) spectroscopy, solid-state nuclear magnetic resonance (NMR), circular dichroism (CD), thermal gravimetric analysis (TGA) and vibrating sample magnetometer (VSM). DLS analysis revealed an average microcapsule size of 1.3 μm (Figure 4a). Zeta-potential measurements showed a value of +62.3 mV, confirming the presence of cationic imidazolium groups from IL-NH2-BMB on the surface of the chiral microcapsules (Supporting Information, Figure S5). Infrared spectroscopy identified the presence of the bis(mandelato)borate anion and characteristic polyurea bands (Figure 4b). Specifically, the strong peak at 3316 cm^−1^ corresponded to N–H stretching bands of urea groups, while peaks at 1735 cm^−1^ were attributed to the carbonyl group stretching band of the bis(mandelato)borate anion. Peaks at 1671 cm^−1^ and 1599 cm^−1^ were assigned to free and hydrogen-bonded C=O stretching bands of the urea group, respectively. Additionally, peaks between 1239 and 1107 cm^−1^ indicated overlapping stretching bands of B-O and C-O bonds.

**Figure 5:**
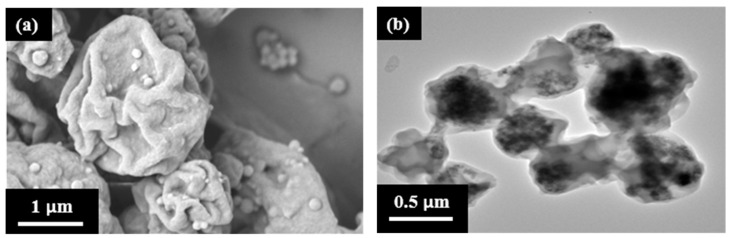
(**a**) SEM image and (**b**) TEM image of magnetically separable chiral (polyionic liquid) microcapsules prepared using DMSO as polar solvent and the R-isomer of IL-NH_2_-BMB.

SEM analysis revealed a spherical squeezed morphology of the microcapsules, suggesting a core–shell structure (Figure 5a). Furthermore, TEM analysis confirmed the presence of MNPs-IL-C4 nanoparticles within the cores of the microcapsules (Figure 5b).

**Figure 6:**
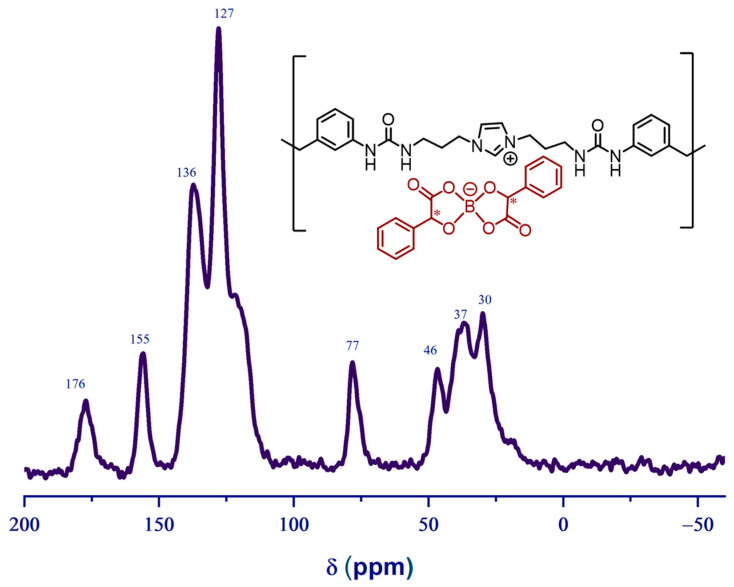
^13^C CP-MAS NMR spectrum of chiral (polyionic liquid) microcapsules prepared using DMSO as polar solvent and the R-isomer of IL-NH_2_-BMB. The asterisk (*) refers to a chiral center.

Further evidence of the formation of chiral poly(ionic liquid) microcapsules was obtained through ^13^C CP-MAS NMR analysis (Figure 6). The analysis was conducted on a sample prepared using DMSO as the polar solvent and the R-isomer of IL-NH_2_-BMB in the absence of MNPs-IL-C4. The spectrum revealed broad peaks ranging between 28 and 50 ppm, clearly indicating the presence of all carbons from the methylene groups of IL-NH_2_-BMB and the methylene group of PAPI 27. The peak at 77 ppm corresponds to the benzylic carbon atom of the bis(mandelato)borate anion connected to an oxygen atom, confirming the presence of the chiral anion within the microcapsule shell. Peaks between 120 and 150 ppm represent the carbons of the imidazolium group, the phenyl groups of the bis(mandelato)borate anion, and the phenyl groups of the polyurea. The peak at 155 ppm is attributed to the carbon atom of the carbonyl group of the urea groups, while the peak at 176 ppm is assigned to the carbon atom of the carbonyl group of the bis(mandelato)borate anion.

**Figure 7:**
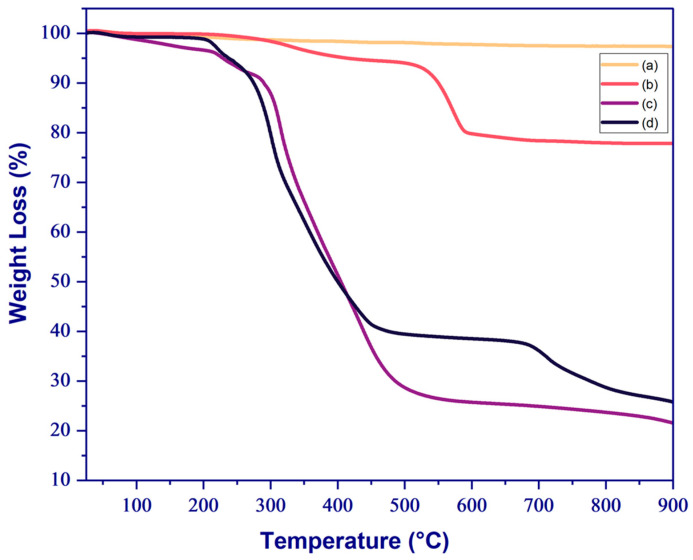
TGA curves of (**a**) bare magnetic nanoparticles, (**b**) MNPs-IL-C4, (**c**) magnetically separable chiral poly(ionic liquid) microcapsules, and (**d**) chiral poly(ionic liquid) microcapsules.

Thermal gravimetric analysis (TGA) was employed to assess both the thermal stability and organic content of the chiral PU-MNPs microcapsules, as depicted in Figure 7. The analysis was conducted under a nitrogen (N_2_) atmosphere, with temperatures ranging from 25 °C to 900 °C, at a heating rate of 10 °C per minute. The bare magnetic nanoparticles exhibited a minor weight loss of 0.5% between 101 °C and 185 °C, primarily due to the desorption of volatile solvents. For the MNPs-IL-C4, two stages of weight loss were observed, between 304 °C and 591 °C, resulting in a total loss of 22.14%, which is attributed to the decomposition of the ionic liquid groups attached to the surface of the magnetic nanoparticles. The weight loss profile of the magnetically separable chiral microcapsules (polyionic liquid) revealed a single stage of decomposition, with a total weight loss of 74%. Conversely, the microcapsules without encapsulated MNPs exhibited a two-step decomposition, resulting in a total weight loss of 78%. The residual percentages in both cases were attributed to either magnetite nanoparticles or non-decomposable species generated during heating, such as boron trioxide. The results from TGA indicate that the existence of magnetite nanoparticles accelerates the degradation of the chiral microcapsules. ICP analysis was used to determine the loading of magnetite nanoparticles within the chiral microcapsules. After dissolving the magnetite in concentrated hydrochloric acid (12 M), the results revealed that the chiral capsules contained 16.2% (*w*/*w*) magnetite.

**Figure 8:**
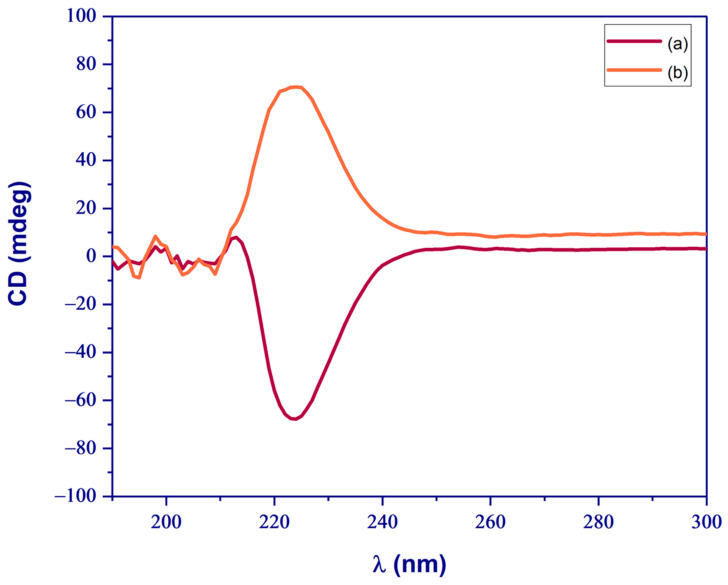
Circular dichroism (CD) spectrum of (**a**) magnetically separable chiral poly(ionic liquid) microcapsules containing R-isomer of bis(mandelato)borate anion and (**b**) magnetically separable chiral poly(ionic liquid) microcapsules containing S-isomer of bis(mandelato)borate anion.

CD measurements were conducted to confirm the chirality of the magnetic chiral poly(ionic liquid) microcapsules which were fabricated using either the R- or S-isomer of IL-NH_2_-BMB (Figure 8). These measurements were carried out using 100 ppm of microcapsules dispersed in methanol. The spectra indicate that the microcapsules exhibited signals akin to the IL-NH_2_-BMB monomers employed in their preparation. Specifically, microcapsules synthesized with the R-isomer of IL-NH_2_-BMB displayed a negative signal at 222 nm (Figure 8a), whereas those prepared with the S-isomer showed a positive signal at the same wavelength (Figure 8b). Notably, there was a blue shift in the wavelength absorption of the chiral microcapsules compared to the absorption wavelength of IL-NH_2_-BMB, which can likely be attributed to solvent effects.

**Figure 9:**
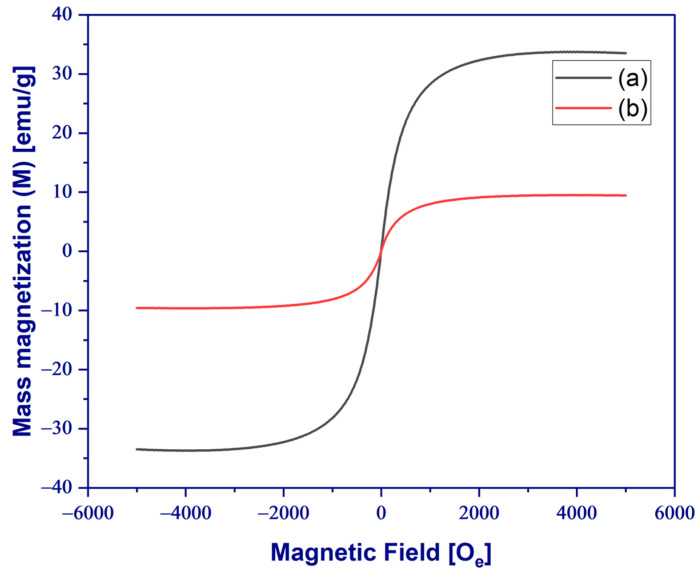
The VSM curves of (**a**) MNPs-IL-C4 and (**b**) magnetically separable chiral poly(ionic liquid) microcapsules containing R-isomer of bis(mandelato)borate anion.

The magnetic properties of MNPs-IL-C4 and the magnetically separable chiral (polyionic liquid) microcapsules prepared using the R-isomer of IL-NH2-BMB were investigated through VSM measurements at room temperature. Figure 9 presents the magnetization curves for both materials. The results reveal no hysteresis in the magnetization curves, along with no significant coercivity or remanence indicating a superparamagnetic behavior. The saturation magnetization of MNPs-IL-C4 is 33.49 emu/g, while that of the magnetically separable chiral microcapsules is 9.44 emu/g. The reduced saturation magnetization of the microcapsules is attributed to the presence of non-magnetic polymeric material.

### 3.4. Enantioselective Adsorption of Tryptophan Enantiomers by Magnetically Separable Chiral Poly(ionic liquid) Microcapsules

The chiral recognition capabilities of the magnetically separable chiral poly(ionic liquid) microcapsules were examined, with tryptophan acting as the model molecule. Experimental conditions involved a racemic mixture of tryptophan dissolved in water at a concentration of 0.49 mM, combined with 200 mg of the chiral microcapsules. Enantioselective adsorption experiments utilized chiral poly(ionic liquid) microcapsules containing either the R- or S-isomer of bis(mandelato)borate anion in their shell. The adsorbed enantiomers on the microcapsules were evaluated using CD spectroscopy. Calibration curves of various concentrations for each enantiomer (Figures S7 and S8, Supporting Information) were employed to determine the percentage of enantiomeric excess (%ee) [48] in the solutions after selectively adsorbing the preferred enantiomer from the racemic mixture.

When chiral poly(ionic liquid) microcapsules with the R-isomer of bis(mandelato)borate anion were mixed with the racemic mixture of tryptophan, and the optical activities were measured over time to determine which enantiomer was preferentially adsorbed and in what quantity, it was observed that the signal of the D-Trp enantiomer increased over time (Figure S9, Supporting Information). This suggests selective adsorption of L-Trp onto the chiral capsules. Conversely, chiral microcapsules with the S-isomer of bis(mandelato)borate anion showed an increase in the L-Trp signal over time (Figure S10, Supporting Information), indicating preferential adsorption of D-Trp onto these microcapsules.

**Figure 10:**
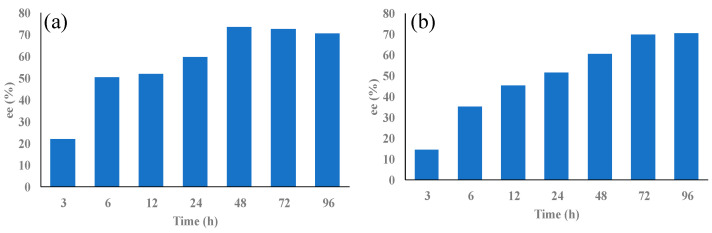
The enantiomeric excess at different time intervals for the solutions measured after mixing racemic tryptophan with chiral poly(ionic liquid) microcapsules containing (**a**) the S-isomer of the bis(mandelato)borate anion and (**b**) the R-isomer of the bis(mandelato)borate anion.

The enantiomeric excess (ee) of the solutions was analyzed after their interaction with chiral microcapsules, with CD measurements taken at 3, 6, 12, 24, 48, 72, and 96 h intervals. As shown in Figure 10, the results reveal interesting trends. When using chiral microcapsules containing the S-isomer of bis(mandelato)borate anion, an initial ee of 22% was observed after 3 h. The ee gradually increased, reaching a maximum of 73% after 48 h (Figure 9a). Extending the mixing time to 72 and 96 h did not increase the enantiomeric excess, with a slight decrease in %ee observed, indicating that equilibrium in the adsorption–desorption process was achieved after 48 h. In contrast, using chiral microcapsules with the R-isomer of bis(mandelato)borate anion resulted in a lower initial ee of 14% after 3 h (Figure 9b), but the enantiomeric excess steadily increased, peaking at 70% after 96 h of mixing.

**Figure 11:**
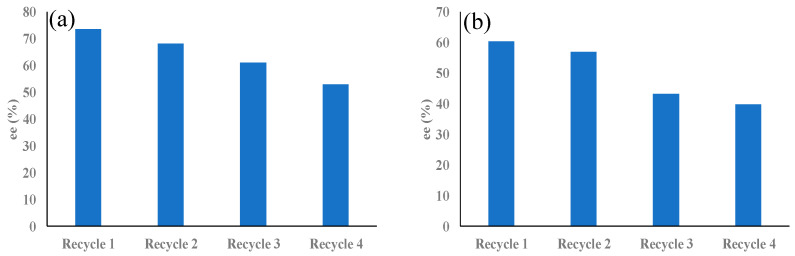
Recycling of the chiral poly(ionic liquid) microcapsules containing (**a**) the S-isomer and (**b**) the R-isomer of the bis(mandelato)borate anion in the enantioselective adsorption of racemic tryptophan.

The reusability of the chiral microcapsules in the enantioselective adsorption of racemic tryptophan was assessed over four recycling cycles (Figure 11). Both types of chiral microcapsules were tested, with ee measured after 48 h of mixing with racemic tryptophan. After each cycle, the microcapsules were recovered using an external magnetic field and washed three times with water to remove the adsorbed tryptophan. The results indicated that chiral microcapsules containing the S-isomer or R-isomer of bis(mandelato)borate anion showed a slight decrease in ee after the second cycle, with a more pronounced reduction observed after the third and fourth cycles (Figure 11).

## 4. Conclusions

We have developed a new method for preparing magnetically separable chiral polymeric microcapsules, utilizing interfacial polymerization and oil-in-oil emulsification processes. This technique involves the initial emulsification of a polar phase containing magnetite nanoparticles modified with ionic liquid groups (IL-C4) and a chiral diamine monomer (IL-NH_2_-BMB) with an imidazolium group and a chiral bis(mandelato)borate anion, into a non-polar phase with an appropriate polymeric surfactant. The process results in the formation of magnetically retrievable chiral poly(ionic liquid) microcapsules which can be applied in the enantioseparation of racemic tryptophan.

Our findings indicate that chiral microcapsules containing the R-isomer of the bis(mandelato)borate anion selectively adsorb L-tryptophan, while those with the S-isomer selectively adsorb D-tryptophan. This method presents a significant advancement in the preparation of chiral materials, offering great potential for applications in asymmetric catalysis, chiral crystallization, and the enantioselective release of racemic active compounds.

## Figures and Tables

**Table 1:** Preparation of magnetically separable chiral poly(ionic liquid) microcapsules under different conditions.

Entry	Configuration of IL-NH_2_-BMB	Polar Solvent	MNPs-IL-C4 (mg)	TEM Images
1	R	DMSO	0	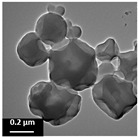
2	R	DMSO	20	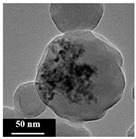
3	R	DMSO	40	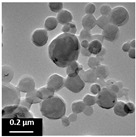
4	R	DMSO	80	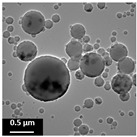
5	R	DMSO	200	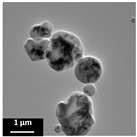
6	S	DMSO	200	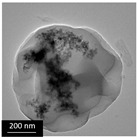
7	R	DMF	20	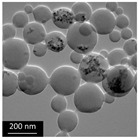
8	R	DMF	40	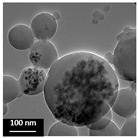
9	R	DMF	80	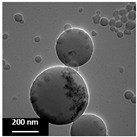
10	R	DMAc	20	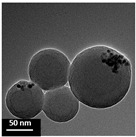
11	R	DMAc	40	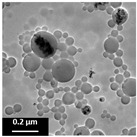
12	R	DMAc	160	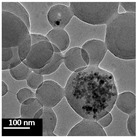

## Data Availability

The original contributions presented in the study are included in the article/Supplementary Materials, further inquiries can be directed to the corresponding author.

## Supplementary Materials

The following supporting information can be downloaded at: https://www.mdpi.com/article/10.3390/polym16192728/s1, Figure S1: Infrared spectrum of MNPs-IL-C4; Figure S2: Particle size distribution of MNPs-IL-C4 in different solvents; Figure S3: TEM image of magnetically separable chiral poly(ionic liquid) microcapsules prepared using DMSO as the polar solvent, 200 mg of MNPs-IL-C4, the R-isomer of IL-NH_2_-BMB and 5% of surfactant ABIL-EM90; Figure S4: TEM image of magnetically separable chiral poly(ionic liquid) microcapsules prepared using DMSO as the polar solvent, 200 mg of MNPs-IL-C4, the R-isomer of IL-NH_2_-BMB and different concentrations of the surfactant Agrimer AL 22; Figure S5: Zeta potential of magnetically separable chiral poly(ionic liquid) microcapsules prepared using DMSO as the polar solvent and 200 mg of MNPs-IL-C4 with the R-isomer of IL-NH2-BMB; Figure S6: Circular dichroism (CD) spectrum of (a) D-tryptophan and (b) L-tryptophan at a concentration of 0.49 mM; Figure S7: (1) Circular dichroism (CD) spectra of L-tryptophan at different concentrations. (2) Calibration curve of L-tryptophan at different concentrations using the CD spectra; Figure S8: (1) Circular dichroism (CD) spectra of D-tryptophan at different concentrations. (2) Calibration curve of D-tryptophan at different concentrations using the CD spectra; Figure S9: CD spectra obtained for solutions formed by mixing chiral poly(ionic liquid) microcapsules containing the R-isomer of the bis(mandelato)borate anion with racemic tryptophan at different time intervals; Figure S10: CD spectra obtained for solutions formed by mixing chiral poly(ionic liquid) microcapsules containing the S-isomer of the bis(mandelato)borate anion with racemic tryptophan at different time intervals; Figure S11: CD spectra for solutions obtained by mixing of chiral poly(ionic liquid) microcapsules containing the R-isomer of the bis(mandelato)borate anion with racemic tryptophan across different cycles; Figure S12: CD spectra for solutions obtained by mixing of chiral poly(ionic liquid) microcapsules containing the S-isomer of the bis(mandelato)borate anion with racemic tryptophan across different cycles; Figures S13–S24: ^1^HNMR and ^13^C NMR spectra.
