# Supplementary material for: Magnetically Separable Chiral Poly(ionic liquid) Microcapsules Prepared Using Oil-in-Oil Emulsions
Source: Polymers (Basel). 2024 Sep 26;16(19):2728. doi: 10.3390/polym16192728 (PMC11478766; doi:10.3390/polym16192728)
Supplement: Supplementary file 1 [file polymers-16-02728-s001.zip › polymers-3160135-supplementary.pdf]

Supporting information

Magnetically Separable Chiral Poly(ionic liquid) Microcapsules Prepared Using Oil-in-Oil Emulsions

Reema Siam[1], Abeer Ali[1] and Raed Abu-Reziq[1,*]

[1] Institute of Chemistry, Casali Center of Applied Chemistry, Center for Nanoscience and Nanotechnology, the Hebrew University of Jerusalem 9190401, Israel.

* Correspondence: Raed.Abu-Reziq@mail.huji.ac.il; Tel.: +972-2-6586097

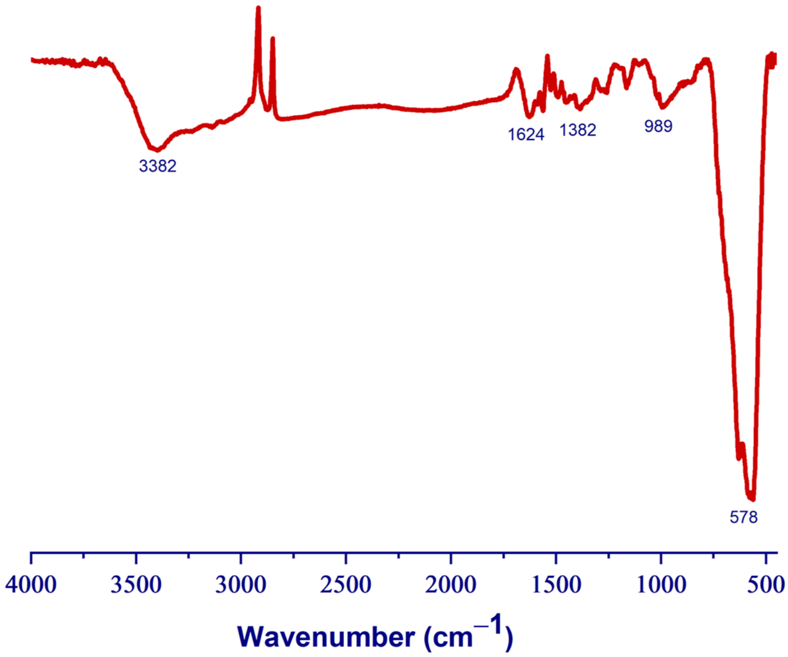

Figure S1. Infrared spectrum of MNPs-IL-C4.

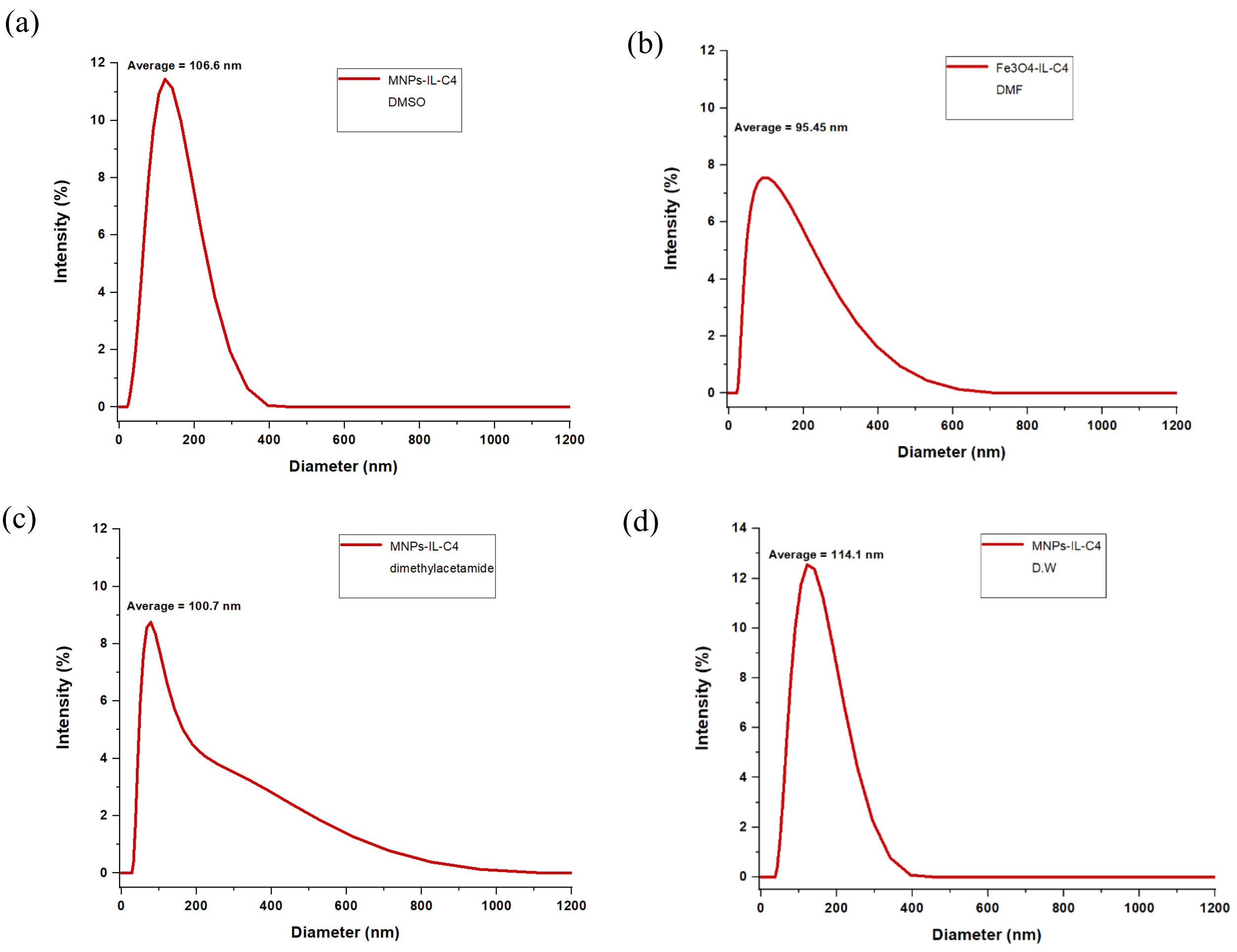

Figure S2. Particle size distribution of MNPs-IL-C4 in different solvents: (a) DMSO, (b) DMF, (c) DMAc, and (d) water.

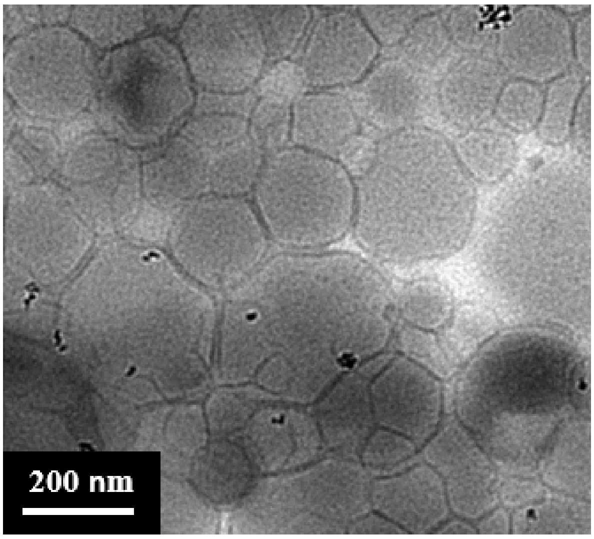

Figure S3. TEM image of magnetically separable chiral poly(ionic liquid) microcapsules prepared using DMSO as the polar solvent, 200 mg of MNPs-IL-C4, the R-isomer of IL-NH_2-BMB, and 5% of surfactant ABIL-EM90

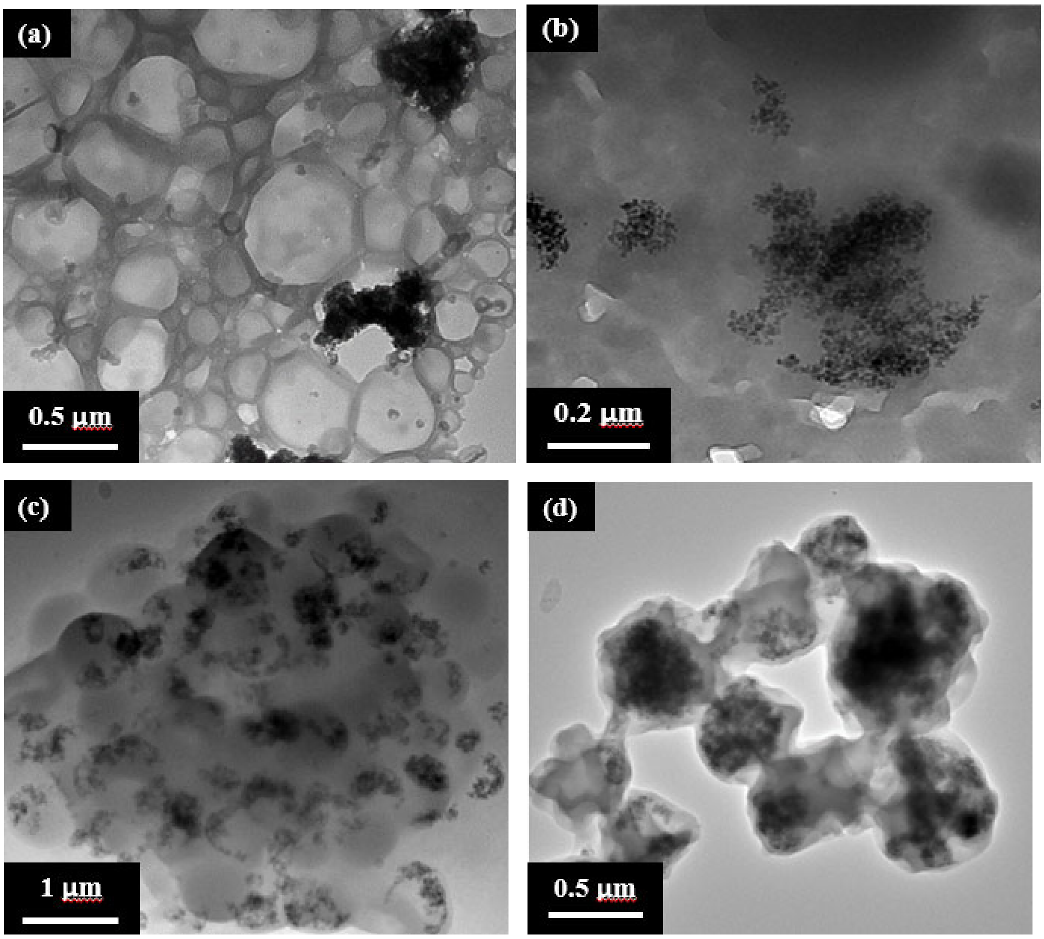

Figure S4. TEM image of magnetically separable chiral poly(ionic liquid) microcapsules prepared using DMSO as the polar solvent, 200 mg of MNPs-IL-C4, the R-isomer of IL-NH_2-BMB, and different concentrations of the surfactant Agrimer AL 22: (a) 1% ratio, (b) 2% ratio, (c) 4% , and (d) 5%.

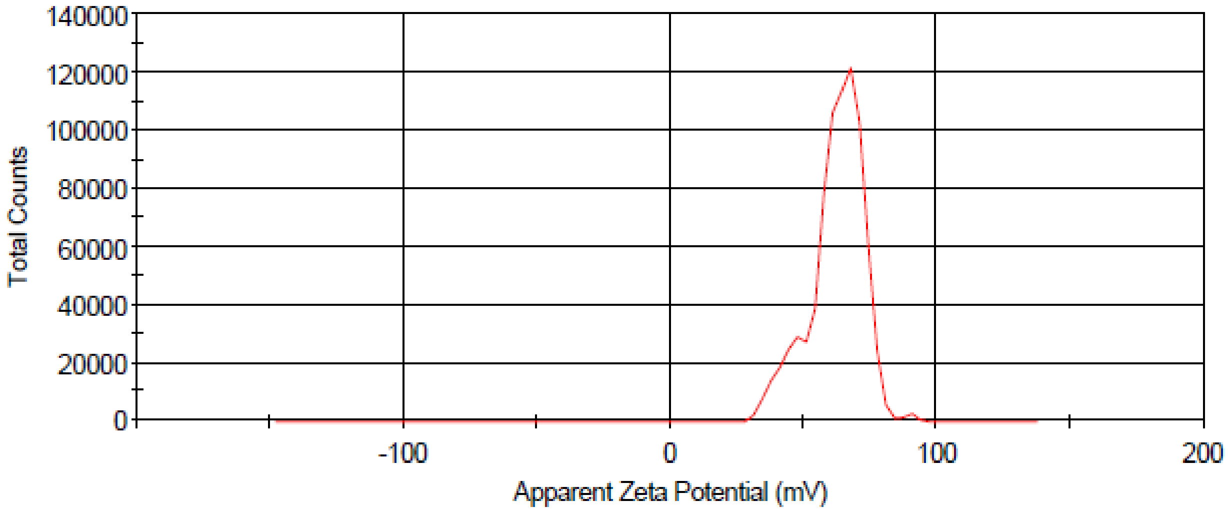

Figure S5. Zeta potential of magnetically separable chiral poly(ionic liquid) microcapsules prepared using DMSO as the polar solvent and 200 mg of MNPs-IL-C4 with the R-isomer of IL-NH_2-BMB.

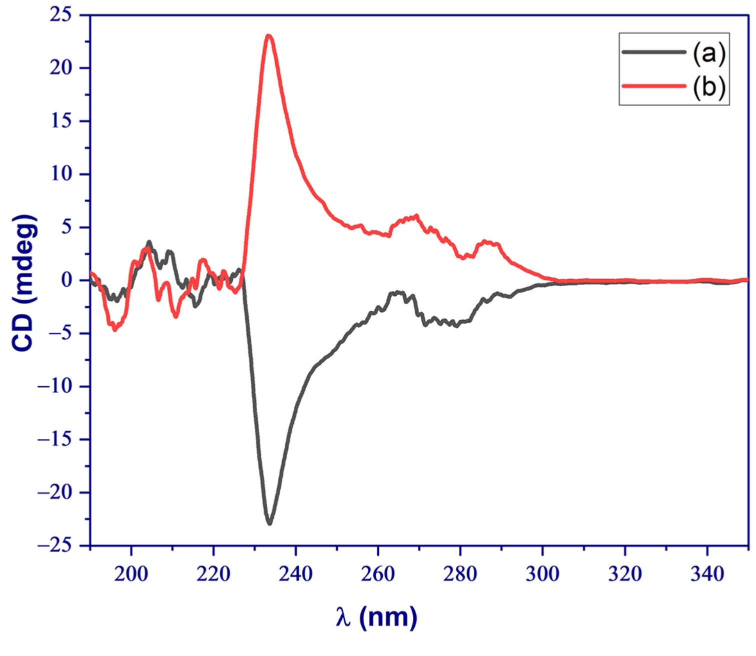

Figure S6. Circular dichroism (CD) spectrum of (a) D-tryptophan and (b) L-tryptophan at a concentration of 0.49 mM.

(1)

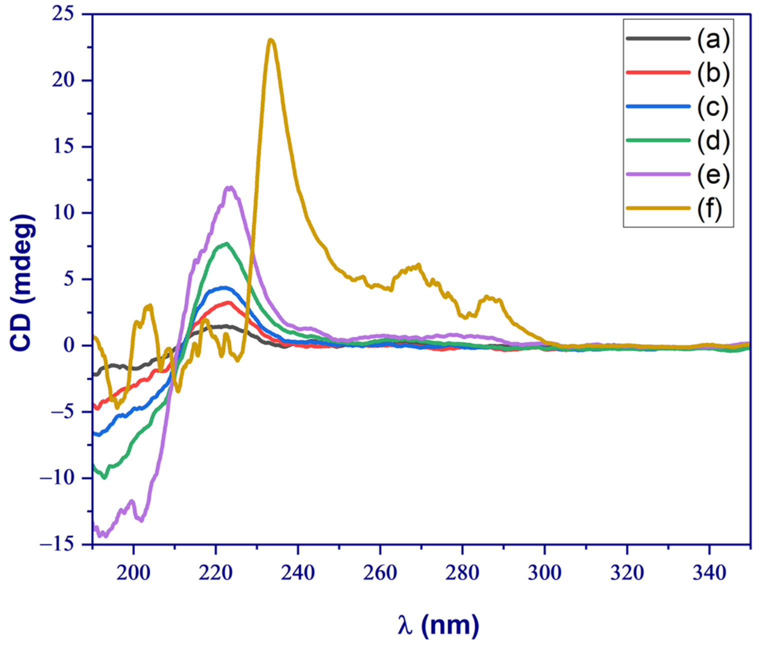

(2)

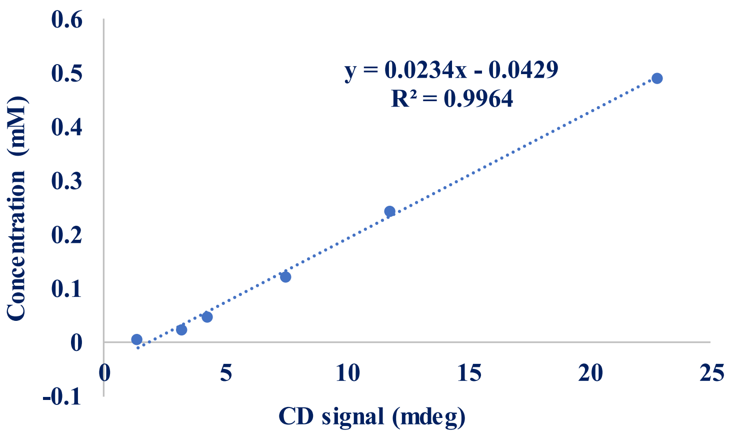

Figure S7. (1) Circular dichroism (CD) spectra of L-tryptophan at different concentrations: (a) 0.0049 mM, (b) 0.0244 mM, (c) 0.049 mM, (d) 0.122 mM, (e) 0.245 mM, and (f) 0.49 mM. (2) Calibration curve of L-tryptophan at different concentrations using the CD spectra.

(1)

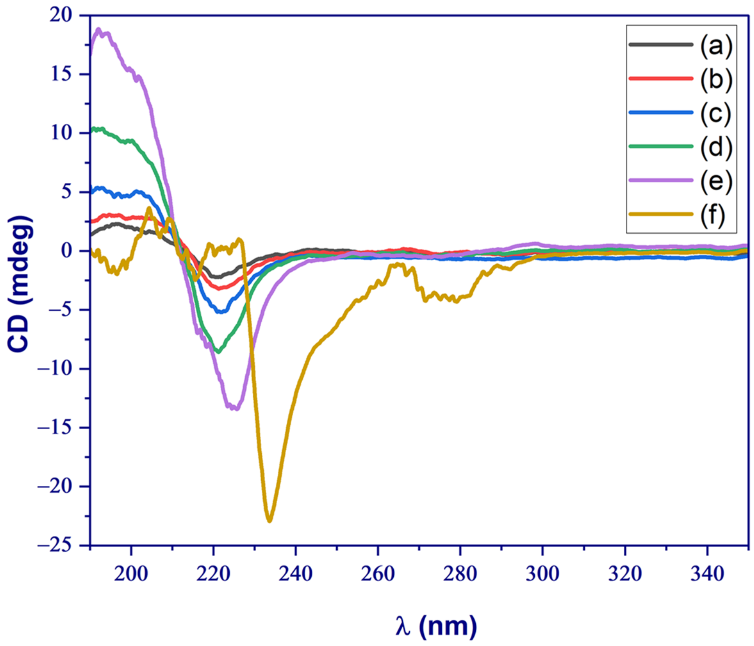

(2)

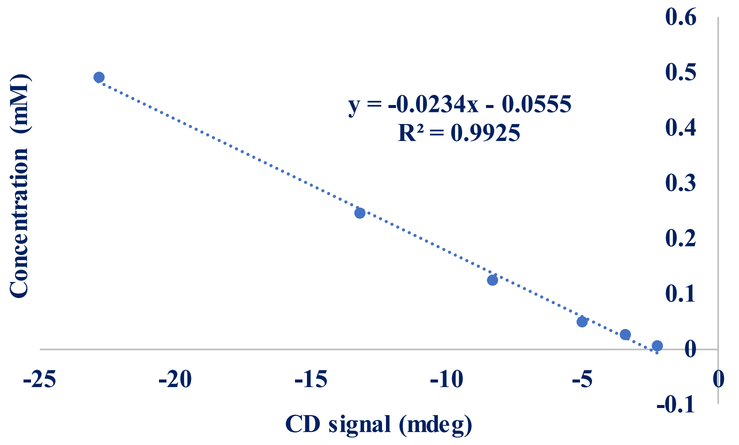

Figure S8. (1) Circular dichroism (CD) spectra of D-tryptophan at different concentrations: (a) 0.0049 mM, (b) 0.0244 mM, (c) 0.049 mM, (d) 0.122 mM, (e) 0.245 mM, and (f) 0.49 mM. (2) Calibration curve of D-tryptophan at different concentrations using the CD spectra.

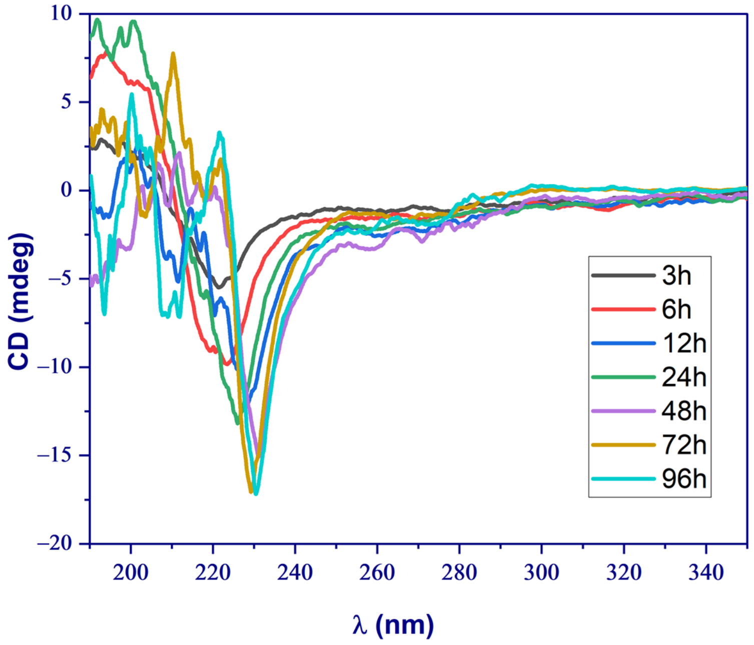

Figure S9. CD spectra obtained for solutions formed by mixing chiral poly(ionic liquid) microcapsules containing the R-isomer of the bis(mandelato)borate anion with racemic tryptophan at different time intervals.

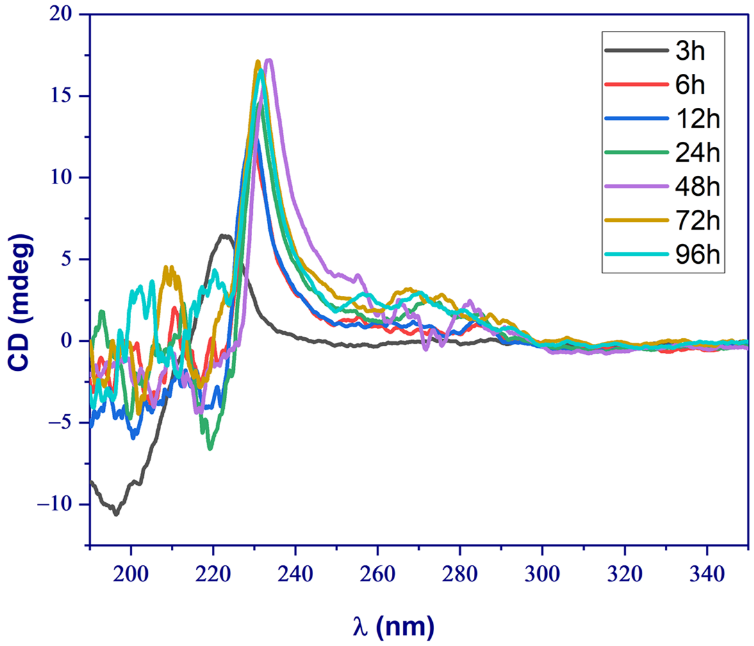

Figure S10. CD spectra obtained for solutions formed by mixing chiral poly(ionic liquid) microcapsules containing the S-isomer of the bis(mandelato)borate anion with racemic tryptophan at different time intervals.

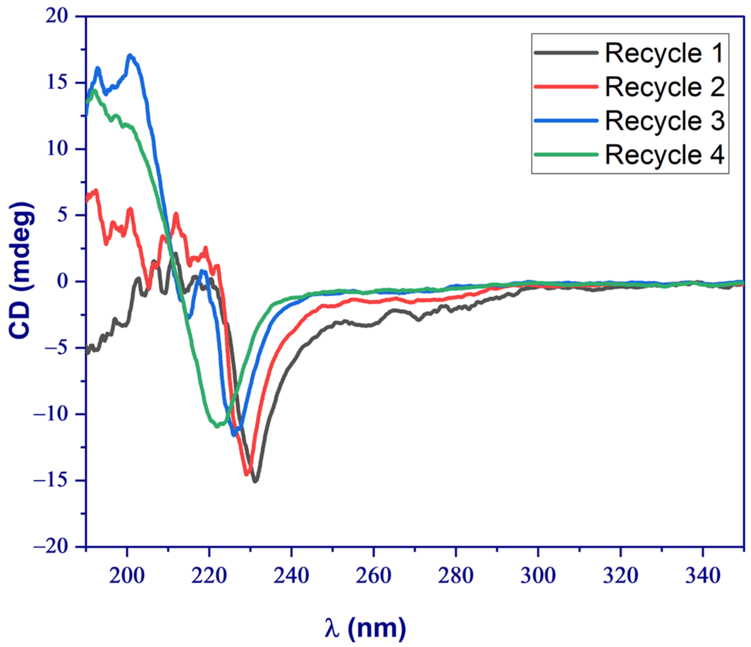

Figure S11. CD spectra for solutions obtained by mixing of chiral poly(ionic liquid) microcapsules containing the R-isomer of the bis(mandelato)borate anion with racemic tryptophan across different cycles.

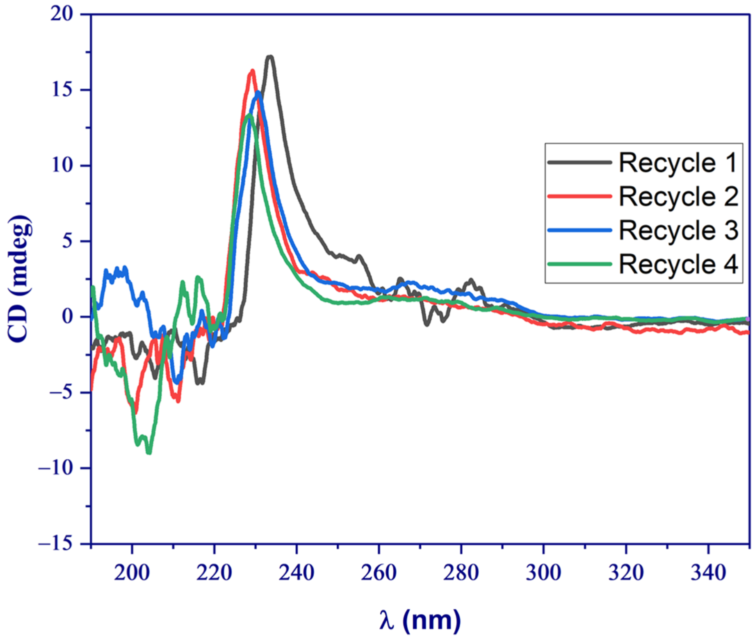

Figure S12. CD spectra for solutions obtained by mixing of chiral poly(ionic liquid) microcapsules containing the S-isomer of the bis(mandelato)borate anion with racemic tryptophan across different cycles.

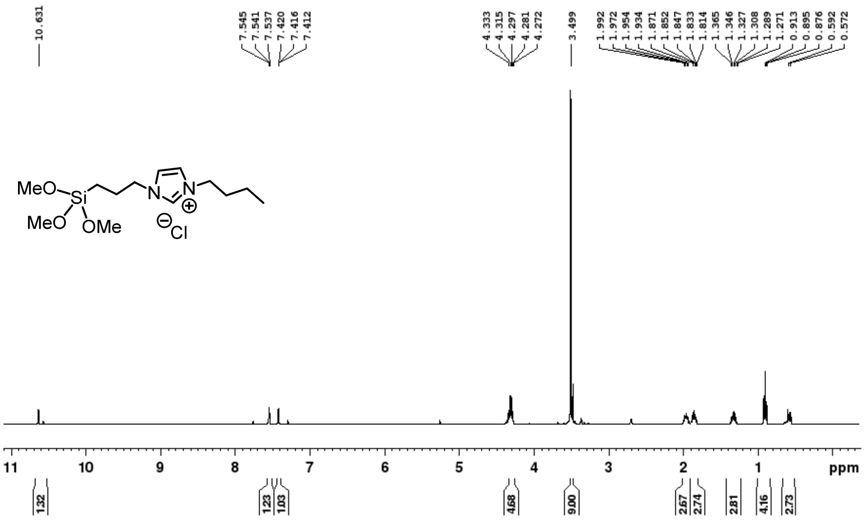

Figure S13. ^{1}H NMR spectrum of 1-butyl-3-(3-(trimethoxysilyl)propyl)-1*H*-imidazol- 3-chloride (IL-C4).

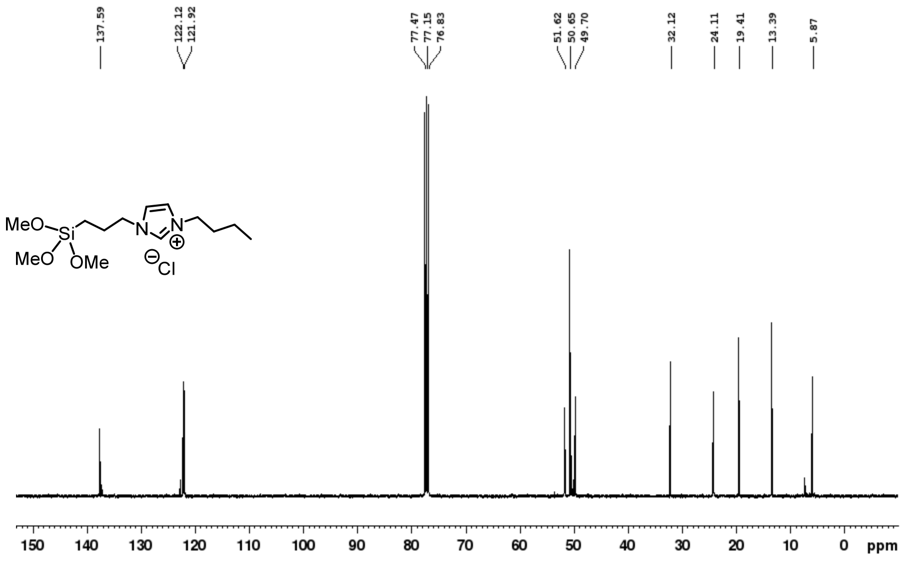

Figure S14. ^{13}C NMR spectrum of 1-butyl-3-(3-(trimethoxysilyl)propyl)-1*H*-imidazol- 3-chloride (IL-C4).

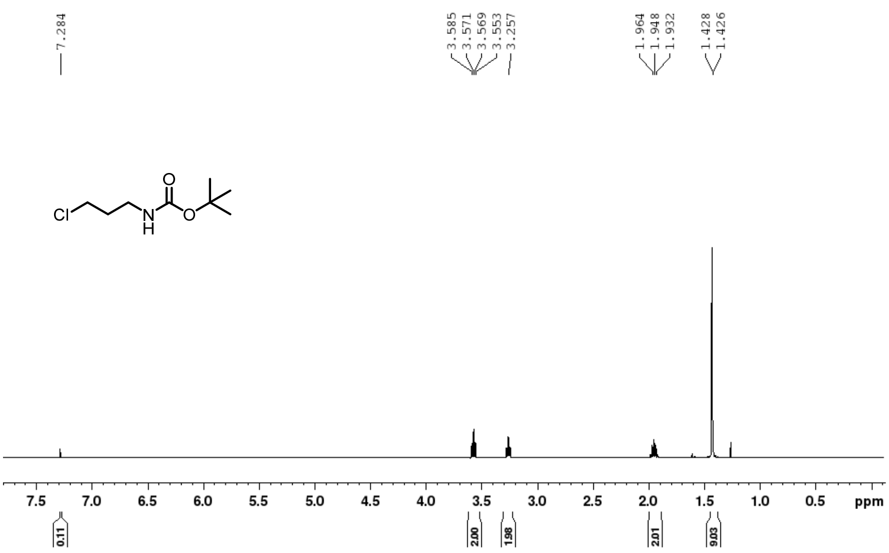

Figure S15. ^{1}H NMR spectrum of *tert*-butyl N-(3-chloropropyl)carbamate.

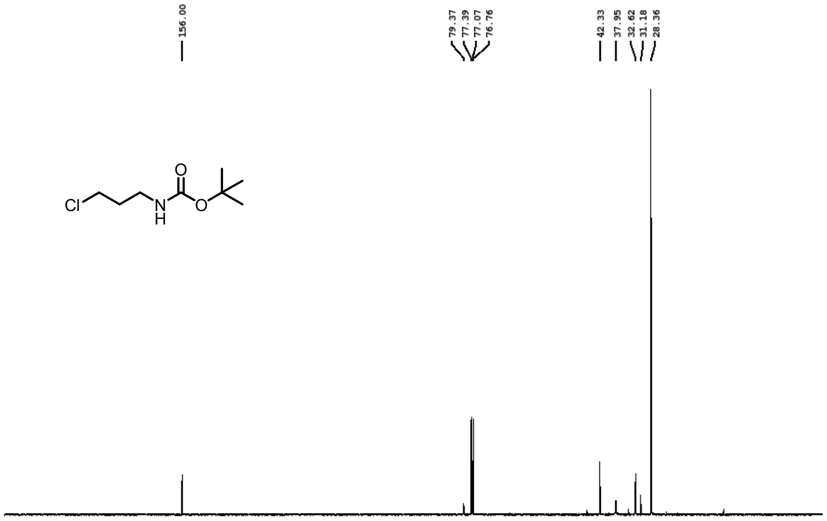

Figure S16. ^{13}C NMR spectrum of *tert*-butyl N-(3-chloropropyl)carbamate.

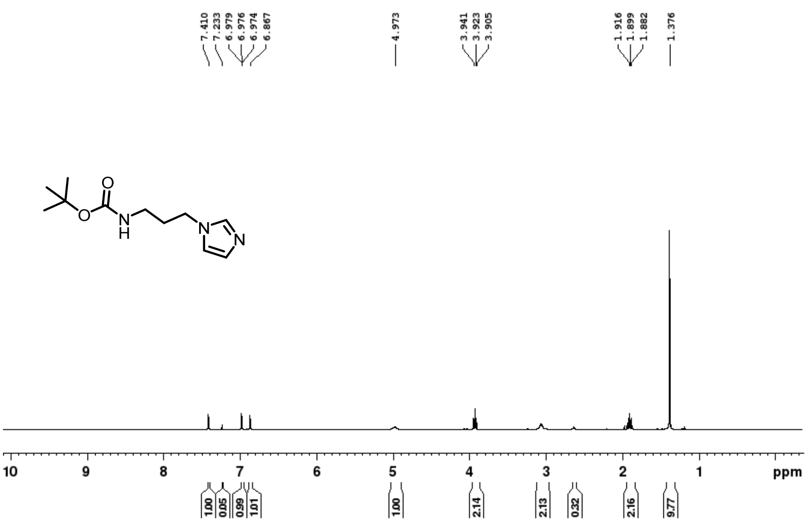

Figure S17. ^{1}H NMR spectrum of *tert*-butyl (3-(1*H*-imidazol-1-yl)propyl)carbamate.

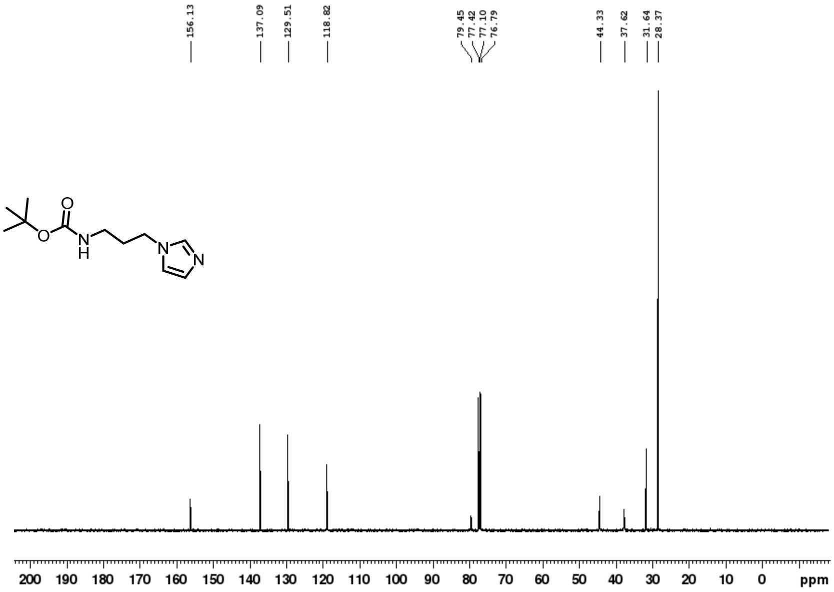

Figure S18. ^{13}C NMR of *tert*-butyl (3-(1*H*-imidazol-1-yl)propyl)carbamate.

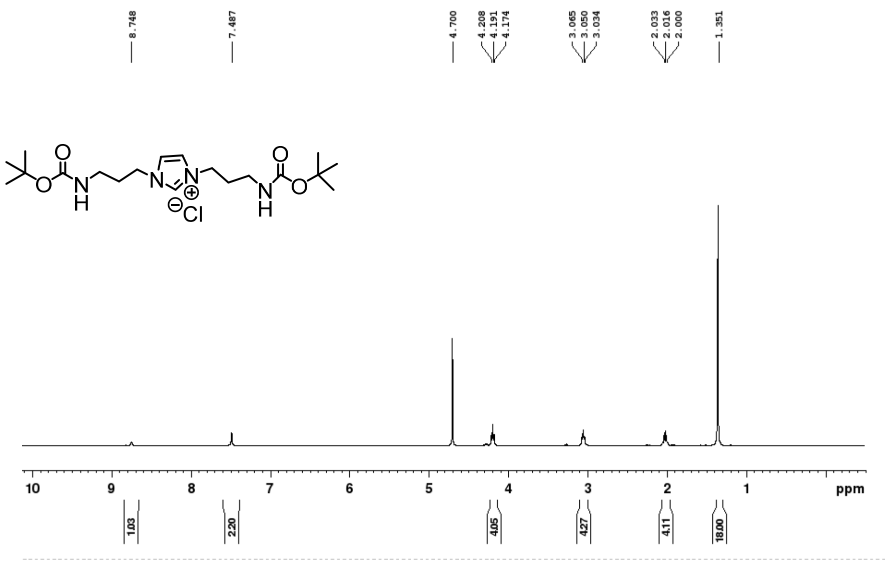

Figure S19. ^{1}H NMR spectrum of 1,3-bis(3-((tert-butoxycarbonyl)amino)propyl)-1*H*-imidazol-3-ium chloride (BAPIC).

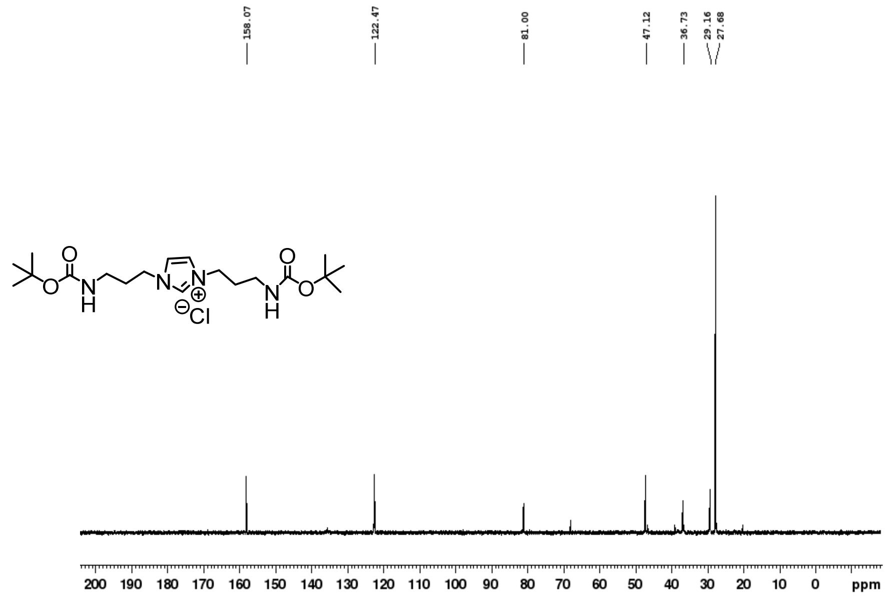

Figure S20. ^{13}C NMR spectrum of 1,3-bis(3-((tert-butoxycarbonyl)amino)propyl)-1*H*-imidazol-3-ium chloride (BAPIC).

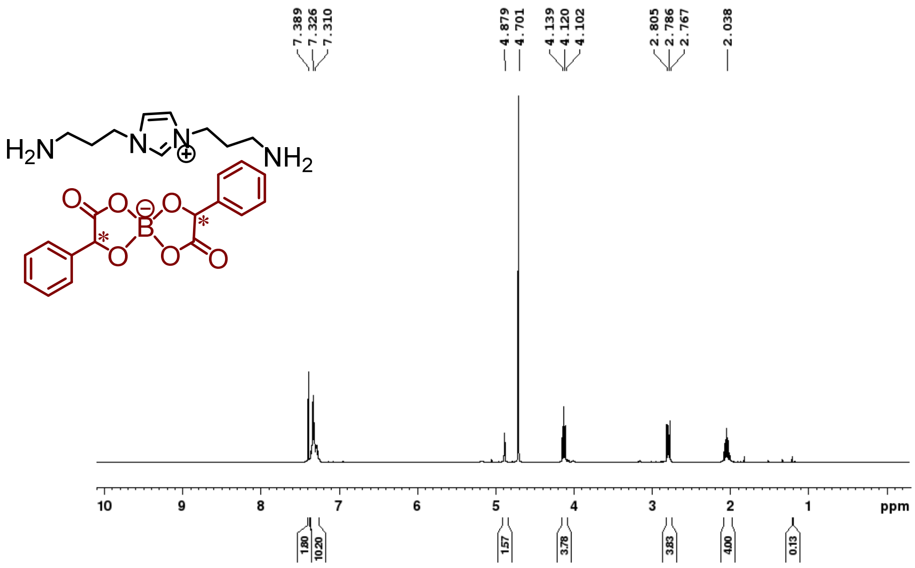

Figure S21. ^{1}H NMR spectrum of 1,3-bis(3-aminopropyl)-1*H*-imidazol-3-ium-2,7-dioxo-3,8-diphenyl-1,4,6,9-tetraoxa-5-boraspiro[4.4]nonan-5-uide (IL-NH_2-BMB) in D_2O. The asterisk (*) refers to a chiral center.

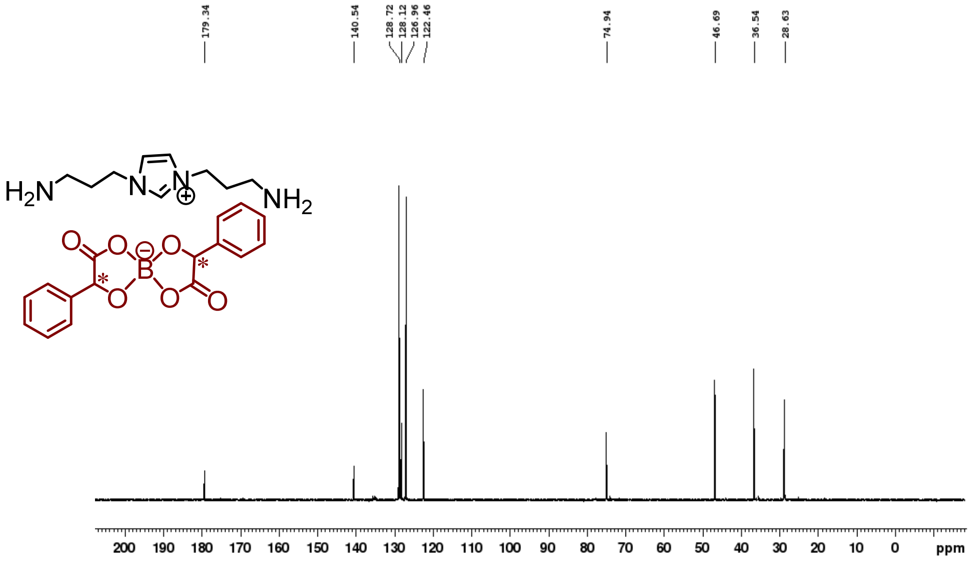

Figure S22. ^{13}C NMR spectrum of 1,3-bis(3-aminopropyl)-1*H*-imidazol-3-ium-2,7-dioxo-3,8-diphenyl-1,4,6,9-tetraoxa-5-boraspiro[4.4]nonan-5-uide (IL-NH_2-BMB) in D_2O. The asterisk (*) refers to a chiral center.

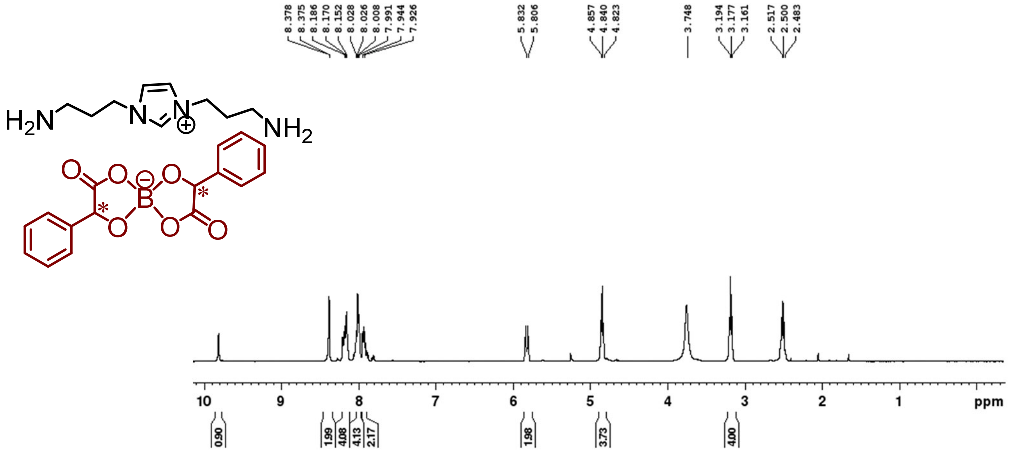

Figure S23. ^{1}H NMR spectrum of 1,3-bis(3-aminopropyl)-1*H*-imidazol-3-ium-2,7-dioxo-3,8-diphenyl-1,4,6,9-tetraoxa-5-boraspiro[4.4]nonan-5-uide (IL-NH_2-BMB) in DMSO-d_6. The asterisk (*) refers to a chiral center.

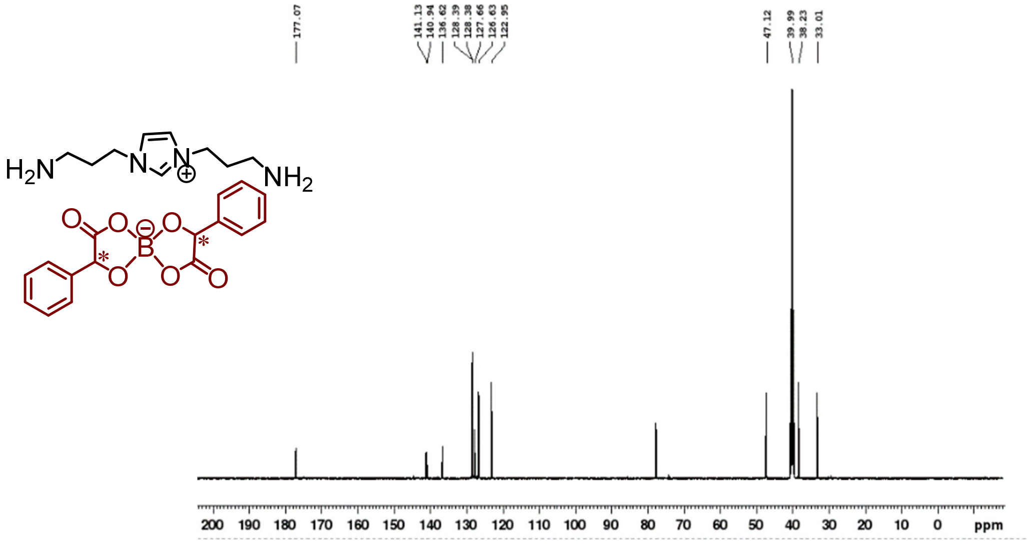

Figure S24. ^{13}C NMR spectrum of 1,3-bis(3-aminopropyl)-1*H*-imidazol-3-ium-2,7-dioxo-3,8-diphenyl-1,4,6,9-tetraoxa-5-boraspiro[4.4]nonan-5-uide (IL-NH_2-BMB) in DMSO-d_6. The asterisk (*) refers to a chiral center.
